# Mix-method toolbox for monitoring greenhouse gas production and microbiome responses to soil amendments

**DOI:** 10.1016/j.mex.2024.102699

**Published:** 2024-04-16

**Authors:** Késia Silva Lourenço, Afnan Khalil Ahmad Suleiman, Agata Pijl, Mauricio R. Dimitrov, Heitor Cantarella, Eiko Eurya Kuramae

**Affiliations:** a Microbial Ecology Department, Netherlands Institute of Ecology (NIOO), Droevendaalsesteeg 10, Wageningen 6708, PB, The Netherlands; b Soils and Environmental Resources Center, Agronomic Institute of Campinas (IAC), Av. Barão de Itapura 1481, Campinas 13020-902, SP, Brazil; c Soil Health group, Bioclear Earth B.V., Rozenburglaan 13, Groningen 9727 DL, The Netherlands; d Ecology and Biodiversity, Institute of Environmental Biology, Utrecht University, Utrecht, The Netherlands

**Keywords:** Next generation sequencing (NGS), Gas measurement, Sustainability, Climate change, Global warming, Nitrous oxide, Methane, Carbon dioxide, Fungal community, Bacterial community, Dynamics of soil microbiome, Soil disturbances, Resistance and resilience, Time-series analyses, Co-occurrence of fungal and bacteria, Quantitative real-time PCR (QPCR), Vinasse, Organic residues, Interdisciplinary methods used to assess the impact of organic residues on various environmental components (soil, atmosphere, and plants). The methods are:I. Soil DNA sequencing and quantitative real-time PCR (qPCR),II. Bioinformatic and statistical analyses,III. Greenhouse gases (GHG) sampling and GHG chromatography analysis,IV.Plant biomass determination

## Abstract

In this study, we adopt an interdisciplinary approach, integrating agronomic field experiments with soil chemistry, molecular biology techniques, and statistics to investigate the impact of organic residue amendments, such as vinasse (a by-product of sugarcane ethanol production), on soil microbiome and greenhouse gas (GHG) production. The research investigates the effects of distinct disturbances, including organic residue application alone or combined with inorganic N fertilizer on the environment. The methods assess soil microbiome dynamics (composition and function), GHG emissions, and plant productivity. Detailed steps for field experimental setup, soil sampling, soil chemical analyses, determination of bacterial and fungal community diversity, quantification of genes related to nitrification and denitrification pathways, measurement and analysis of gas fluxes (N_2_O, CH_4_, and CO_2_), and determination of plant productivity are provided. The outcomes of the methods are detailed in our publications (Lourenço et al., 2018a; Lourenço et al., 2018b; Lourenço et al., 2019; Lourenço et al., 2020). Additionally, the statistical methods and scripts used for analyzing large datasets are outlined. The aim is to assist researchers by addressing common challenges in large-scale field experiments, offering practical recommendations to avoid common pitfalls, and proposing potential analyses, thereby encouraging collaboration among diverse research groups.

• Interdisciplinary methods and scientific questions allow for exploring broader interconnected environmental problems.
• The proposed method can serve as a model and protocol for evaluating the impact of soil amendments on soil microbiome, GHG emissions, and plant productivity, promoting more sustainable management practices.
• Time-series data can offer detailed insights into specific ecosystems, particularly concerning soil microbiota (taxonomy and functions).

## Graphical abstract

**Figure:**
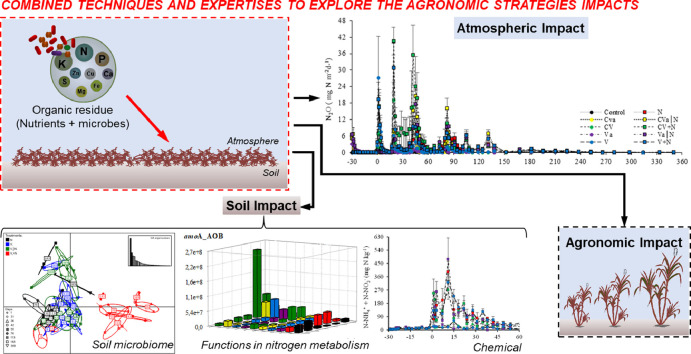

Specifications table

**Table:** 

Subject area:	Agricultural and Biological Sciences
More specific subject area:	Microbial ecology Soil microbiology Environmental science Soil fertility Soil science Plant production systems Climate change Global warming
Name of your method:	Here, we outline the interdisciplinary methods used to assess the impact of organic residues on various environmental components (soil, atmosphere, and plants). The methods are: I. Soil DNA sequencing and quantitative real-time PCR (qPCR): these techniques are employed to evaluate the dynamics of soil microbial community composition and quantify the N cycle genes associated with N_2_O production; II. Bioinformatic and statistical analyses: both methods are essential to explore the changes in the soil microbiome composition and function; III. Greenhouse gases (GHG) sampling and GHG chromatography analysis: in this step gases are collected in static chambers and analyzed in a gas chromatograph with an electron capture detector for N_2_O determination and a flame ionization detector for CO_2_ and CH_4_ determination; IV. Plant biomass determination: Biometric method developed for sugarcane crop.
Name and reference of original method:	NA
Resource availability:	All amplicon sequencing data are deposited at European Nucleotide Archive (ENA) and publicly accessible under project number PRJEB30929 (ITS sequences) and PRJEB25676 (16S rRNA sequences).

**Method details**

This study aims to provide researchers with a practical interdisciplinary mix-methods toolbox to explore the impact of agronomic strategies in mitigating environmental challenges. The methods investigate how single and multiple consecutive pulse disturbances resulting from the application of soil amendments such as organic residue, inorganic N fertilizer, or their combination, affect the soil microbial community structure and functions, gene abundances related to the nitrogen cycle, greenhouse gases (GHG) emissions, and plant productivity (Fig. 1). Here we describe approaches used in four different publications [[1], [2], [3], [4]] (Fig. 2), which includes field experiment setup, GHG sampling and analysis, soil sampling and soil chemical analyses, microbial molecular analysis by qPCR and next generation sequencing of bacterial and fungal phylogenetic markers, as well as bioinformatic and statistical data analyses. These publications also offer valuable insights, including guidance on experiment planning and sampling efforts.

**Fig. 1:**
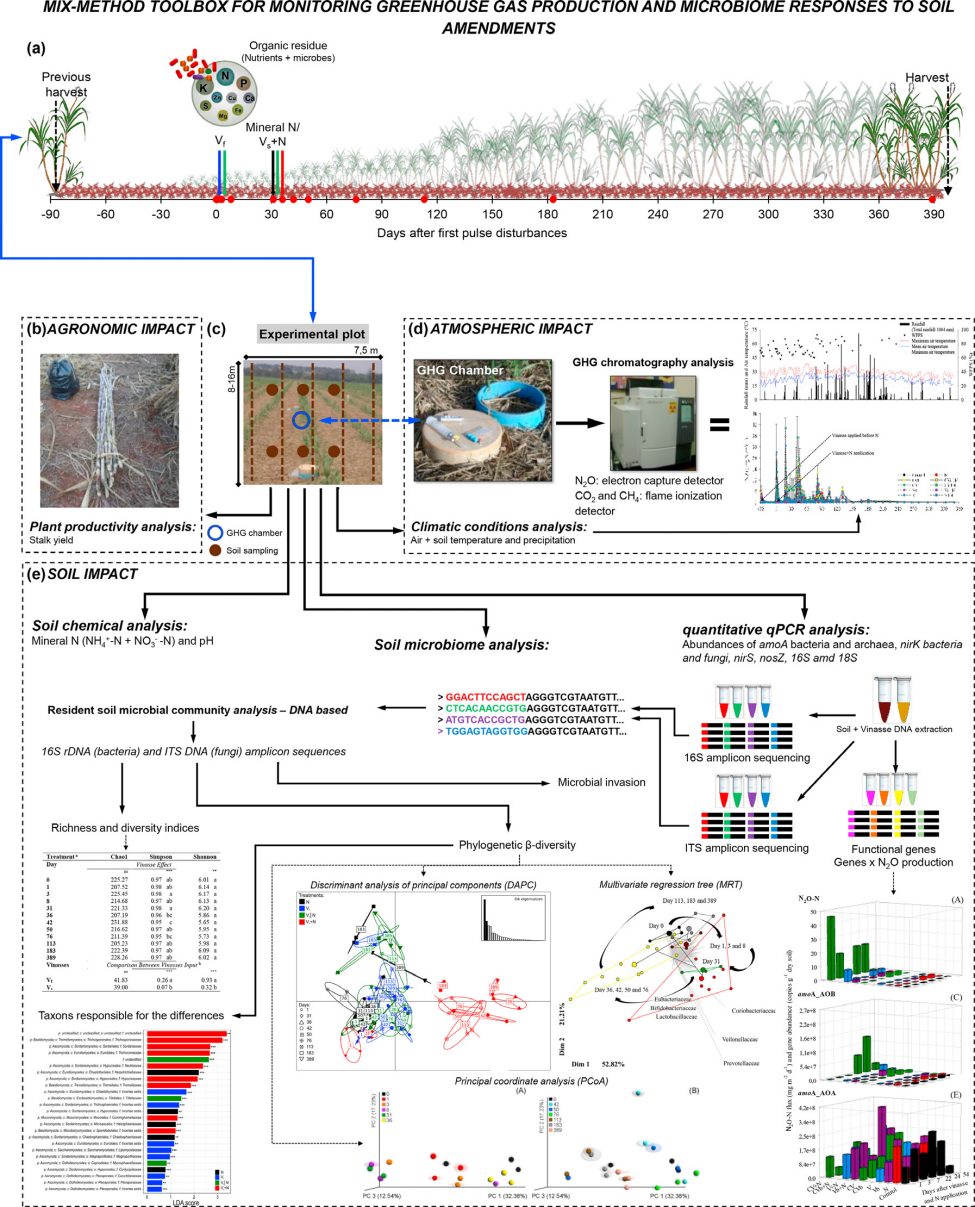
(a) On-site treatment scheme at sugarcane field experiment. Time of application of mineral fertilizer N (ammonium nitrate) and vinasse to sugarcane and sugarcane harvest time. The treatments were as follows: V_f_, vinasse applied at day 0; N, inorganic fertilizer ammonium nitrate applied at day 30; V_f_│N, vinasse applied at day 0 and ammonium nitrate applied at day 30; and V_s_+N, vinasse plus ammonium nitrate applied only at day 30. The red points represent the different sampling time points, the dashed arrows represent the period of sugarcane harvest, and the colors of the bold arrows represent the different treatments: N, black; Vf, blue; Vf│N, green; and Vs+N, red. (b) Schematic overview of the experiment and results of the impact of vinasse and inorganic fertilizer on plant growth (stalk yield). (c) Schematic overview of the experiment plots with the greenhouse gas (GHG) chambers and soil sampling method. (d) Schematic overview of the GHG chambers and results of the impact of vinasse and inorganic fertilizer on GHG emissions and climatic conditions. (e) Schematic overview of the experiment and results of the impact of vinasse and inorganic fertilizer on biological processes linked to nitrous oxide (N_2_O) emission and soil bacterial and fungal communities.

**Fig. 2:**
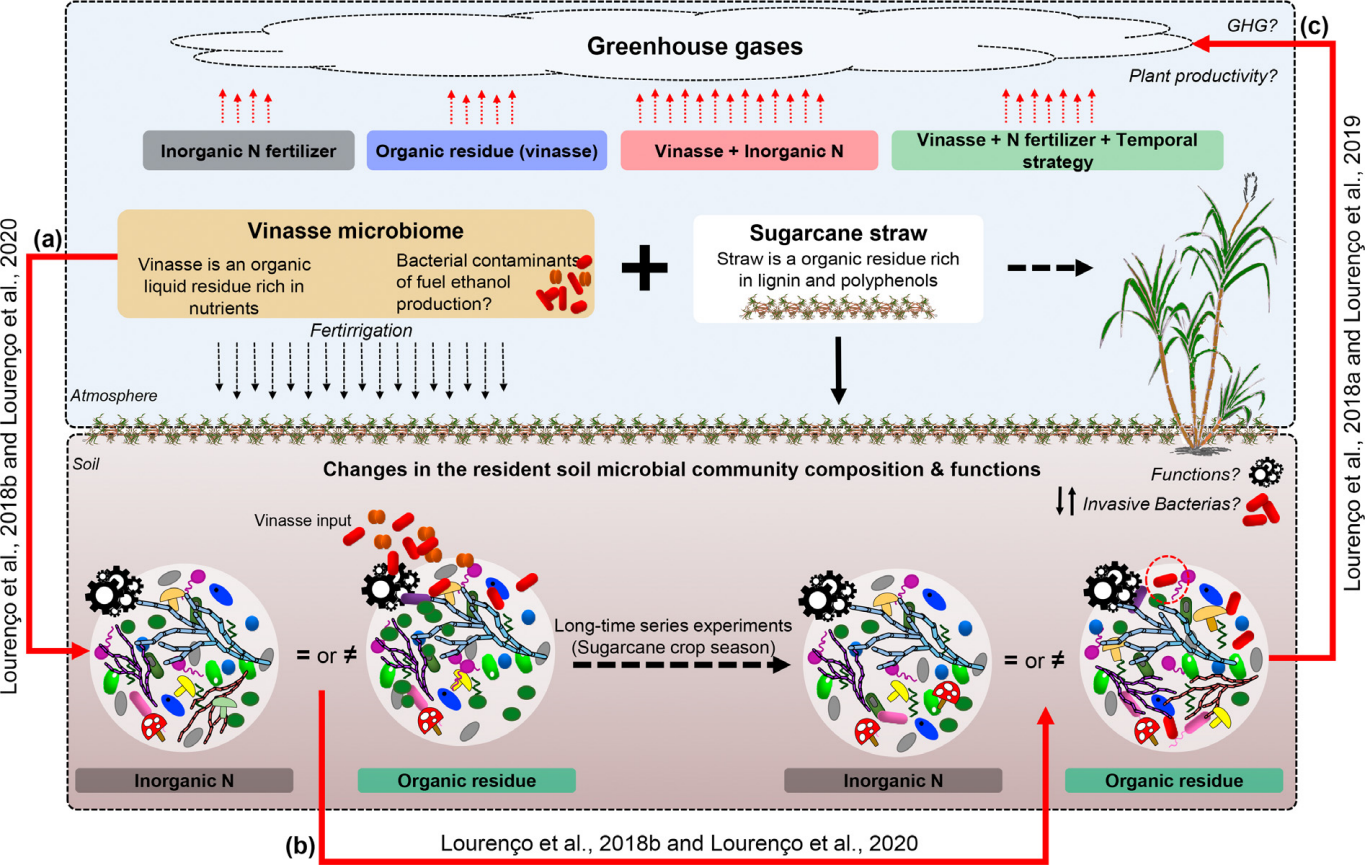
Schematic overview of published papers utilizing the interdisciplinary approach, integrating agronomic field experiments with soil chemistry, molecular biology techniques and statistics to investigate the impact of vinasse (by-product of sugarcane ethanol production) on soil microbiome and greenhouse gas (GHG) production. The research investigated the effects of distinct disturbances, including organic residue application alone or combined with inorganic N fertilizer on the (a) soil bacteria and fungi communities and their interaction, (b) the microbiome dynamics throughout the whole season (390 days), and (c) the impact on the gene abundances related to the nitrogen cycle, greenhouse gases (GHG) emissions, and plant productivity.

In particular, we sampled soils from sugarcane cultivation as our model soil-plant ecosystem. We introduced disturbances in this ecosystem by applying organic residue, both with and without inorganic fertilizer. The organic residue, applied either 30 days before, after, or along with the mineral N fertilizer, was vinasse, a significant by-product of the sugarcane biofuel industry. Vinasse contains microbes, nutrients, and organic matter and is commonly recycled as fertilizer [5]. Researchers can use this toolbox with mix-methods for various systems, including different plant species, both no-till and conventional tillage practices, and agropastoral farming systems, among others.

## Field experiment setup

### Weather conditions

The research questions play a crucial role as they determine the number of treatments, the number of experiments, what variables to evaluate, and methods to use to answer these questions. In the study example all experiments were designed with the same main purpose: to understand the impact of organic residues on GHG production and evaluate different temporal strategies of organic residue application to decrease N_2_O emissions. If researchers are evaluating the role of fertilization in the system, similar to the study example, they have to consider that fertilization occurs under varying climatic conditions in different regions. Therefore, researchers need to determine the most representative conditions for their study. Different weather conditions can lead to variations in gas emissions and microbial community responses to fertilizer addition. In this context, the study utilized three distinct experiments conducted in the southeast of Brazil, where mineral and organic fertilization in sugarcane takes place between April and November, spanning three seasons: fall to spring (fall and winter-dry/spring-wet). Due to these varying climatic conditions, the experiments were conducted in different seasons: R1 during the rainy season of the 2013/2014 cycle, D1 during the dry season of the 2014/2015 cycle, and R2 during the rainy season of the 2014/2015 cycle [2]. One of the objectives was to evaluate how climatic conditions influence the effects of fertilization in the sugarcane system. Rainy and dry seasons were defined based on precipitation levels in the first three months after the fertilizer application.

### Replicates and experimental design

The cost of each analysis, the number of variables, and the available labor all play a crucial role in determining the minimum number of replicates per treatment needed. For meaningful and statistically reliable results, a minimum of three replicates is required for all analyses (gas, microbial and chemical soil analysis). After deciding the number of treatments and replicates, researchers must visit the designated area for the experiment in advance to determine the optimal allocation of plots and treatments. This assessment is crucial before initiating the experiment. Factors such as land slope, rainwater runoff, areas prone to rainwater accumulation, sunlight direction, the presence of trees, ants, termites, and other animals, as well as proximity to urban areas, need to be considered. These factors will determine whether the experiment should be organized into blocks or if a completely randomized setup is feasible. The size of the plots can also vary based on this information. In our case example, although the sugarcane fields are relatively uniform, there were slight inclinations. Therefore, the area was divided into four blocks to minimize variability. The experiments were structured in a randomized block design comprising ten treatments and four blocks (totalling 40 plots).

### Field conditions characteristics

Once the experiment design and number of replicates are determined, it is crucial to characterize the field conditions, especially the soil type and its characteristics. Factors such as nutrient availability, texture, organic matter content, pH, and drainage capacity can vary significantly between different soil types. As a result, understanding and accounting for these variations are essential when designing experiments to accurately assess GHG emissions and microbial dynamics in agricultural and environmental research. This information is fundamental when conducting experiments such as crop fertilization. Researchers conducting soil physical and chemical analyses should seek reliable laboratories in their regions or even send soil samples abroad if necessary. It is crucial to use globally accepted methods well-documented in the scientific literature. Recommended soil analysis methods can vary between regions. These methods are often calibrated and validated for the specific regions to ensure their accuracy and reliability. Additionally, certain analytical techniques may not be universally available worldwide. It is important to note that results can vary between methods, making comparisons challenging. In this study example, the three experiments were conducted in Brazil; based on this, methods recommended for Brazilian soils in the São Paulo State were utilized for soil physical [6] and soil chemical [7] characterization. A total of 20 random soil subsamples (0–20 cm soil layer) were collected from each block (two per plot) for detailed soil characterization. Researchers in different countries or regions should decide on the most suitable methods for their specific conditions.

Another crucial parameter is soil density and water retention capacity. To measure these parameters, soil samples were collected from four trenches (one per block), each measuring 0.6 m long, 0.5 m wide, and 0.5 m in depth, excavated with a shovel. Given the homogeneous nature of the area, no variation in the soil density was found. Soil density was determined using a metal ring (∼100 cm^3^) with an intact soil core inside (see Figure S1 for an example). Additionally, the water retention curve was established by determining soil moisture levels under saturated conditions, at 6 kPa, 10 kPa, 30 kPa, 100 kPa, and 1500 kPa. This information allowed for the calculation of the water balance in the experimental areas.

### Crop stage and sampling period

Each crop undergoes various stages and cultural management practices, making the selection of the sampling period and plant stage for evaluation a significant challenge. Different crops have specific requirements that researchers must meticulously consider. In the context of sugarcane, for example, it can regrow up to five times after the initial harvest, known as the ratoon cycle. Researchers need to align their sampling periods with these distinct plant stages to ensure accurate and relevant data collection. In our study with sugarcane, due to the intensity of variability in sampling (throughout the year), it was challenging to find different areas close to each other with similar soil and environmental conditions in the same sugarcane stage. Consequently, it was decided to conduct all experiments on the same farm, independently of the sugarcane stage, since the cultural management is the same for all of them, except for the sugarcane plant stage when the mineral N is incorporated into the soil. The experiments were set up with sugarcane in the third, fourth and second ratoon stages in R1, D1, and R2, respectively. This approach maintains consistency in the experiments while acknowledging the complexities of the sugarcane growth cycle. Since the goal was to compare the effect of different fertilizer management practices the ratoon stage is not an issue.

### Crop residues

Another crucial factor is the presence of biomass on the soil surface, which is especially important in experiments evaluating GHG emissions and microbial activity. Researchers can use metal frames to determine the amount of crop residues such as straw on the soil; the frame's size can vary depending on the area between plants. For instance, in coffee plantations, there are often many leaves under the trees, while the interrow spaces typically have minimal organic matter. In contrast, in our example with sugarcane plantations study, mechanical harvesting disperses straw evenly across the field. In the study, crop residue was measured using a 1 m^2^ metal frame (Figure S2). All crop residue inside the frame was collected and weighed; 8 samples were randomly collected in the experiment (2 per block). Sub-samples were dried in an oven at 40  °C until a constant weight was achieved for dry weight measurement. Using the amount of crop residue inside each frame and considering the moisture content, the total dry residue for the entire plantation can be calculated. Following the recommendation to choose the methods for soil sample analysis, the dried crop residue samples should be ground into a fine powder. Later, the samples can be measured for macro (N, P, K, Ca, S, and Mg) and micronutrients (Fe, Mn, Zn, Cu, and B). In the study, the chemical composition of the residue was determined using methods recommended in São Paulo state, Brazil, a common method used worldwide [8].

### Treatments selection

The treatments in our experiment example consisted of various combinations of organic residues, including concentrated vinasse (CV) and non-concentrated vinasse (V), with or without inorganic N fertilizers. These vinasses and inorganic N were applied at different time intervals: vinasses were applied before (30 days) – anticipated (V_a_│N and CV_a_│N), simultaneously (*V* + *N* and CV+N) or after the inorganic fertilizer (27 days) – postponed (N│V_p_ and N│CV_p_). All experiments had control treatments without N or vinasses (CV or V). The treatments are shown in Table 1. The goal was to evaluate the treatments with organic residue plus N (four treatments in total). However, due to the combined application of organic and inorganic fertilizer, proper controls with each fertilizer alone were added. Based on this, six more treatments were necessary to isolate the effects of the background, mineral N fertilizer, and each organic residue used. In the example studies, the goal was to carefully evaluate each of the treatments and add the needed controls to isolate the effects. Researchers should pay attention to the inclusion of proper controls; the absence of appropriate treatments can limit the conclusions drawn from the experiment. For example, in our study, was necessary to have a control without N fertilizer addition to calculate the N_2_O emission factor (% of N-fertilizer lost as N_2_O). Some studies lack this information making it not applicable for the calculation.

**Table 1:** Overview of the treatments in the sugarcane study example, Time of application and the corresponding nitrogen rate of inorganic fertilizer (N: ammonium nitrate) and vinasse (concentrated vinasse - CV and non-concentrated vinasse –V). Numbers in parenthesis indicate the amount of N, in kg ha^−1^, contained in vinasses and/or N. N was always applied at 100 kg ha^−1^ of N, but the amount of N in vinasse varied with the batch used.

Treatments a	First rainy season (2013/2014)		Dry season (2014/2015)		Treatments	Second rainy season (2014/2015)	
	November 13, 2013	December 13, 2013	July 15, 2014	August 15, 2014		October 14, 2014	November 10, 2014
Control	–	–	–	–	Control	–	–
CV_a_	missed treatment		CV_a_ (30)	–	CV	CV (46)	–
CV	–	CV (48)	–	CV (52)	CV_p_	–	CV_p_ (36)
V_a_	V_a_ (53)	–	V_a_ (51)	–	V	V (74)	–
V	–	V (53)	–	V (89)	V_p_	–	V_p_ (157)
N	–	N (100)	–	N (100)	N	N (100)	–
CV_a_│N	missed treatment		CV_a_ (30)	N (100)	CV+N	CV+N (46+100)	–
CV+N	–	CV+N (48+100)	–	CV+N (52+100)	N│CV_p_	N (100)	CV_p_ (36)
V_a_│N	V_a_ (53)	N (100)	V_a_ (51)	N (100)	*V* + *N*	*V* + *N* (74+100)	–
*V* + *N*	–	*V* + *N* (53+100)	–	*V* + *N* (89+100)	N│V_p_	N (100)	V_p_ (157)

a aVinasse anticipated application (30 days before N fertilization); p: Vinasse postponed application (27 days after N fertilization).

### Fertilizer rates and application method

Fertilization methods differ based on factors like the fertilizer source, plant species, and plant stage. In our study example with sugarcane fields, plants are typically arranged in long rows spaced 1.5 m apart. In contrast, coffee plants are usually arranged in long tree rows spaced between 3.4 to 3.6 m. In small cocoaholder farms, the trees are scattered without a defined organization. Pastures also lack a specific pattern in plant distribution. Researchers should take these diverse arrangements into account when deciding the most appropriate fertilizer application and calculate fertilizer rates accurately. For example, in sugarcane fields, non-concentrated vinasse usually is sprayed over the entire area (10,000 m^2^ ha^−1^). In the study, treatments with V, 100 m^3^ ha^−1^ of V were sprayed over the experimental plot using a motorized pump fit with a flow regulator (Figure S3). It was decided to use the average rate of vinasse in sugarcane plantations in the State of São Paulo, Brazil [9]. The specific organic residue vinasse average rate is based on the K concentration (2 g K_2_O L^−1^) and the K taken up by sugarcane (185 kg K_2_O ha^−1^), plus the cation exchange capacity of a soil. On the contrary, CV in sugarcane fields is strategically applied near the plant rows, covering approximately 16 to 20% of the total area. Consequently, the total CV rate is distributed across only ∼1600 to 2000 m^2^ ha^−1^. Researchers should take this precise information into account when calculating the total amount of CV applied in each plot and within the GHG chambers. Another challenge faced was determining the appropriate CV rates for the study. Vinasse undergoes rapid decomposition, and laboratory K analysis is time-consuming, which is often a constraint in field experiments. Due to these limitations, the researchers opted to use the average concentration employed by the sugar mill. In this study, the volume of CV applied was approximately 5.8 times less than that of V, based on its K content (the mill's average). CV was surface-applied using a calibrated plastic watering can in bands located from 10 to 20 cm from the sugarcane row and it was applied at a rate of 17.2 m^3^ ha^−1^ for all experiments (Figure S4).

Similar to CV, inorganic fertilizers (N, P, and K) are also surface-applied in a band in sugarcane ratoon. The N fertilizer applied rate was 100 kg N ha^−1^, a dose recommended for sugarcane fertilization in Brazil [10]. When deciding on the type of fertilization to use in an experiment, researchers should consider the primary objective. If the goal is to evaluate the N_2_O emissions resulting from different nitrogen fertilizer applications, the only variable that should vary in the system is the nitrogen source. All other factors should remain constant. However, if the experiment aims to assess GHG emissions when different fertilizer management practices are used, the control group should remain unaltered. In our study, the objective was to understand the impact of nitrogen (N) from organic and inorganic sources. Phosphorus was uniformly applied to all plots at a rate of 45 kg P_2_O_5_ ha^−1^ as superphosphate. Potassium was applied to the plots that did not receive vinasse, using KCl, at rates equivalent to the K added through vinasse (from the first vinasse application in each experiment) – 290 K_2_O ha^−1^ in R1, 345 K_2_O ha^−1^ in D1, and 320 kg K_2_O ha^−1^ in R2. Regardless of the inorganic fertilizer used, sugarcane fertilization involves applying fertilizers in a band close to the plants. Therefore, the amount of fertilizer per unit area must be carefully calculated. Based on the literature [11], it was assumed that the fertilized band is approximately ∼25 cm wide, representing 16% of the total area.

### One experiment, multiple research objectives

In large-scale projects, researchers can address a wide range of diverse questions, spanning topics related to plant production, ecology, economics, social factors, environmental concerns, and more. Collaboratively, they can utilize one or more experiments, allowing for a comprehensive perspective on their research topics. This approach provides them with a broader view, enabling a more holistic understanding of the subject matter. Having this in mind, in our study, we used one or all the experiments for the analyses, depending on the question only a subset of treatments were selected. The goal of the first study [2] was to define the best strategy to decrease the N_2_O emissions after the addition of vinasse with and without inorganic fertilization and determine the EF values. To achieve this goal, we used data from all experiments (R1, D1 and R2). For research focused on identifying the main pathways related to N_2_O emissions [1], soil samples from R1 and D1 experiments were selected. For these, soil samples based on the N_2_O emissions were selected for molecular analyses, we chose soil samples from days with high and low N_2_O emissions. In another aspect of the research, the dynamics of bacterial and fungi communities after the application of single and consecutive pulse disturbances and the potential invasiveness of microbes from vinasse were evaluated in one of the experiments, D1. The decision to use experiment D1 was based on the possibility of soil sampling one year after fertilizer and vinasse application. Besides, the research question emerged after the end of the experiment R1 and during the conduction of experiment D1. Due to the importance of V during sugarcane production, only treatments with V were selected for both studies [3,4].

## Greenhouse gases analysis

### Greenhouse gas sampling and analyses

Nitrous oxide (N_2_O) and methane (CH_4_) are the main greenhouse gases emitted during agricultural activities. N_2_O emissions from fertilizer can represent 40% of total GHG emissions in sugarcane ethanol production [12]. In coffee production, this figure is even higher, constituting 67% of total GHG emissions [13]. Monitoring these gases is necessary, especially during sugarcane cultivation, as sugarcane in Brazil is cultivated to produce ethanol, aiming to replace fossil fuels. The most common method to sample GHGs from soils is using static flux chambers [14] (Fig. 1d). These chambers offer several advantages: the technique is relatively inexpensive, easy to adopt, versatile, and adaptable to varying conditions. It also enables the differentiation of emissions across the surface area, allowing for the measurement of gases from different treatments nearby. Flux chambers work by placing an open-bottomed chamber on the ground and measuring gas accumulation in the chamber's headspace over a short time [[14], [15], [16], [17]]. Daily gas emissions are calculated based on measurements taken once a day. To get overall gas emissions, measurements from the chambers are combined over specific intervals throughout the experiment, which usually lasts a few months [18].

In this study, round PVC (Polyvinyl chloride) static chambers were utilized (Fig. 1d, Figure S5). These chambers were constructed using PVC pipes with a diameter of 30 cm. The pipes were cut, and the final height of the chambers was 20 cm (30 cm in diameter x 20 cm in height). One end of the chamber was designed to allow a perfect fit for the lid, while the other end was shaped like a knife, enabling the chamber to be easily inserted into the ground. Round chambers stand out as a top choice due to their circular design, guaranteeing a uniform gas distribution inside and thereby ensuring precise measurements. This shape minimizes edge effects, enhancing the reliability of the data collected. Additionally, round chambers exhibit adaptability across diverse terrains and can be effortlessly deployed in different environments without requiring significant modifications. The shape of the lids can vary based on their intended purpose. Some lids may include a fan to assist in air homogenization within the chamber, while others may have an opening designed to accommodate a thermometer. However, each chamber lid must have two openings, each equipped with a valve: one for gas sampling and the other to control the internal air pressure (Fig. 1d). In the study, the chamber lids were made with PVC pipe caps. These caps were perforated in two places, and valves were inserted into the openings.

During the chamber installation, it is advisable to avoid hammering directly on the chamber edges, as it could lead to breakage. Instead, it is recommended to use a piece of wood to cushion the impact (Figure S5). Not only does this method ensure a more uniform insertion of the chamber into the ground, but it also minimizes disturbance to the surrounding environment. GHG chamber should be installed with a wood hammer, avoiding hitting the edges and chamber breakage, at a minimum depth of 5 cm into the soil. In the study chambers were inserted at a minimum depth of 5 cm into the soil and positioned 10 cm away from the sugarcane rows (Figure S5). The chambers were installed at least one week before the application of treatments to minimize the impact of soil disturbance. In the experiments, the chambers were set up 21 days before the application of fertilizer and vinasse in R1 (October 22, 2013), 34 days before in D1 (June 11, 2014), and 7 days before in R2 (October 07, 2014). This timeframe also provided flexibility to adjust the chamber positions in specific adverse conditions, such as water accumulation, sugarcane growth inside the chamber, or the presence of anthills nearby. The chambers must remain open during the experimental period to maintain environmental consistency inside, aligning with the conditions in the surrounding area. The chambers are only closed temporarily during gas sampling procedures. The greenhouse gas (GHG) sampling period should align with the crop cycle. For sugarcane, GHG emissions begin before fertilization and continue until the harvest. In our study, GHG sampling extended for 317, 381, and 290 days in the R1, D1, and R2 experiments, respectively. The final sampling took place one week before the harvest.

Researchers must carefully consider the number of chambers to be installed in the experiment. Factors such as the workforce available for gas sampling, the distance of the experiment from the research center, availability of materials and equipment for gas sampling will determine the maximum number of chambers that can be effectively installed. Moreover, researchers must have a clear understanding of their primary objective. If the aim is to calculate emissions from the entire plantation, chambers should not be limited to the fertilized area but should also be positioned at various locations across the field. The goal is to account for emissions, which are known to be different, such as in the unfertilized areas between crop rows. In the interrow position, there is no fertilizer application, and the root density is smaller than in the fertilized band. In the study, 40 chambers were utilized. Due to the extensive number of treatments and chambers, the control treatment was utilized as the baseline emission (background emission) from the interrow. This decision was based on previous studies conducted within the same research group, which demonstrated comparable emissions between the control rows and the interrow band in sugarcane [19]. Additionally, collecting gas and soil samples from 40 plots is time-consuming, requiring nearly 6 hours per day with the assistance of three individuals.

If the experiment aims to measure the percentage of N fertilizer lost as N_2_O, it is crucial to know the exact amount of fertilizer applied inside each chamber. Therefore, all inputs, whether organic or inorganic fertilizer, applied inside each GHG chamber must be weighed using precise scales. In the case of liquid fertilizer, laboratory graduated cylinders must be used to ensure accurate measurements. In the study, mineral fertilizers were weighed, and the volume of vinasses used was proportional to the field area where they were applied, ensuring a reflection of actual field conditions inside the chambers (Figure S6). To calculate the N fertilizer and CV applied within each GHG chamber, the linear meters where both fertilizers were applied in the field were considered. For instance, considering one hectare equals 10,000 m^2^ and the distance between sugarcane rows is 1.5 m, the N (100 kg ha^−1^ of N) and CV (17.2 m^3^ ha^−1^) rates were applied along 6666.67 linear meters. Inside each chamber, the amount of N applied was 4.50 g of N (equivalent to 14.06 g of NH_4_NO_3_), along with 774 ml of CV. Regarding V, the application was based on the chamber's area (0.07069 m^2^ ). The V rate (100 m^3^ ha^−1^) applied over 10,000 m^2^ , translated to 707 ml of V applied inside each chamber.

In many cases, sampling gases 24 hours a day or even every day is not feasible. To ensure the most accurate data, it is advisable to consistently sample gases at the same time of day. Typically, morning sampling is preferred to avoid the hottest periods. With this principle in mind, gas samples were collected in the mornings before the application of both organic and inorganic fertilizer treatments. The rationale behind pre-fertilization gas sampling was to assess the stability of emissions at the end of the pre-fertilized period. Following fertilizer application, it is advisable to sample gases daily for a week. High emissions are anticipated immediately after fertilizer application, with weather conditions, particularly rainfall, playing a significant role. Missing even one day of sampling can result in the loss of critical emission peaks. Following the first week, it is advisable to sample gases three times per week until noticeable emission peaks are observed. Subsequently, the frequency can be reduced to weekly or even every fifteen days, based on the observed emission patterns and research objectives. This tailored approach ensures a balance between data accuracy and efficient resource utilization throughout the study period. In the study this recommendation was followed, the GHG sampling was based on the N_2_O emission peaks.

The number of samples per chamber and the volume of gas collected can vary, often influenced by financial resources and the availability of personnel for gas sampling. To calculate emission fluxes accurately, it is recommended to conduct at least three sampling per chamber. The duration of each sampling period typically varies; researchers commonly close the chambers for a period ranging from 20 min to 1 hour, during which 3 to 4 gas samples are collected. This approach allows for a balance between obtaining sufficient data points and optimizing available resources. The system to sample the gases is determined by the requirements of the specific equipment being used. Depending on the equipment, the gas samples may need to be kept under pressure to ensure accurate measurements. In the study, fifty milliliters gas samples were collected at 1, 15, and 30 min using syringes, and transferred to pre-evacuated exetainers vials (12 mL) (Figure S7).

In the laboratory, where multiple experiments are conducted simultaneously, researchers need to diligently assess the quality of exetainers vials and septum. Since samples can be stored for extended periods, it's crucial to ensure the integrity of these components. While both exetainers vials and septum can be reused, it's essential to consider that during each sampling event, the septum is perforated by the needle at least three times. Over time, this puncturing reduces the quality of the septum, limiting the duration for which the samples can be stored effectively. If the laboratory capacity is not large enough for samples to be analyzed immediately and extended storage periods are necessary, it is advisable to use a new septum to ensure the integrity of the samples.

In the study, gases were analyzed simultaneously using gas chromatography (Shimadzu GC-2014 model), with an electron capture detector (ECD, 325 °C) for N_2_O determination and a flame ionization detector (FID, 250 °C) for CO_2_ and CH_4_ determinations (Fig. 1d). Before detection, the CO_2_ was reduced to CH_4_ using a methanator (380 °C). Detection and quantitation limits of GC-2014 are 50 ppb for N_2_O, 1 ppm for CO_2_ and 0.1 ppm for CH_4_. The calibration curve used in the study included three standards, containing N_2_O, CO_2_, CH_4_ and synthetic air (99.999% pure): standard 1) 0.313 µmol mol^−1^ of N_2_O, 357.5 µmol mol^−1^ of CO_2_ and 2065 µmol mol^−1^de CH_4_; standard 2) 0.752 µmol mol^−1^ de N_2_O, 1010 µmol mol^−1^ of CO_2_ and 0,999 µmol mol^−1^ de CH_4_; standard 3) 11.240 µmol mol^−1^ of N_2_O, 1531 µmol mol^−1^ of CO_2_ and 3564 µmol mol^−1^ de CH_4_.

**Fig. 3:**
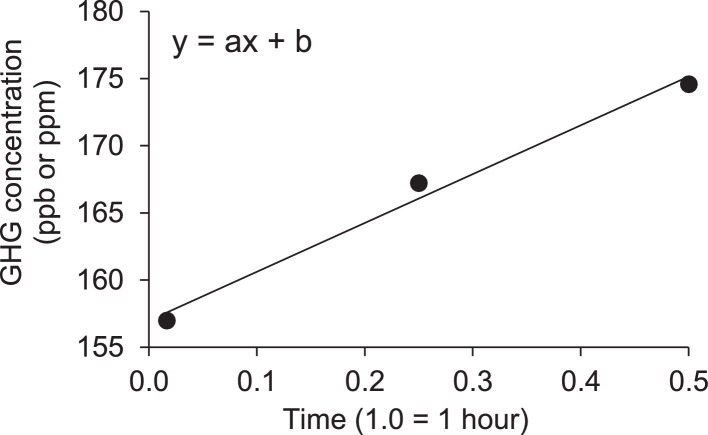
Linear interpolation of the three GHG sampling times. The time variation was equivalent of 0.017 (1 min), 0.250 (15 min) and 0.500 (30 min) of one hour. Where x is the time, b is the GHG concentration in the atmosphere (Y intercept) and a is the slope or GHG gradient.

GHG fluxes are calculated through linear interpolation of the sampling times (Fig. 3) and adjusted by considering the chamber volume, air temperature, and atmospheric pressure. The Ideal Gas Law was employed to calculate the number of moles inside each chamber and the GHG concentration [8]:

(1) $$\text{GHG}\;\text{Flux}=a*\frac{\text{Vm}}{A}*\text{MM}$$

In this context, GHG flux (g m^−2^ h^−1^) represents the emission rate, calculated as a (ΔC/Δt), which signifies the rate of change of gas concentration inside the chamber (measured in ppb h^−1^ or ppm h^−1^ during the incubation time. The 'a' value is derived through interpolation of data from three sampling times (refer to Fig. 3). Vm represents the molar volume of the gas at the sampling temperature (measured in L mol^−1^), MM stands for the molecular mass of N in N_2_O or C in CO_2_ and CH_4_, and A signifies the area of soil covered by the chamber (measured in m2 ).

GHG emission estimates between sampling days are determined through linear interpolation between adjacent sampling dates. Cumulative emissions are calculated by summing the GHG emissions from each sampling day and adding the estimated emissions for the intervening days. Results can also be expressed in total CO_2_ equivalent using the global warming potential (GWP). Summative values of CH_4_ and N_2_O can be multiplied by 27 and 273, respectively, according to the IPCC report [20]. Another approach is to calculate the N_2_O intensity, considering the N_2_O emissions from both treatment and control plots in relation to the crop yield [21].

(2) $$N_{2}O\;\text{intensity}\;=\frac{\text{Cumulative}\;N_{2}O\;\text{emission}\;\left(g\;N\;ha^{-1}\right)\;}{\text{crop}\;\text{yield}\;\left(\text{kg}\;ha^{-1}\right)}$$

Where N_2_O intensity (g N_2_O—N kg^−1^ of stalk) is the amount of N_2_O emitted per kg of crop product, in the study case sugarcane stalk yield.

### Statistical analysis and emission factor for GHG measurements

Firstly, the cumulative emissions must be tested for normality, and if necessary, transformed. In this study, cumulative emissions were transformed using the Box-Cox transformation method. Statistica software, version 10 (Statistica, version 10), was employed for this purpose; however, researchers can use any statistical software available. It is recommended to use the total cumulative emissions from the chamber (mg N_2_O—N m^−2^) (fertilized bands parallel to the crop line) for the statistical analyses. This choice is based on the careful measurement of both fertilizer amounts applied and N_2_O emissions from the chambers, eliminating the need for transformation factors or summation of background emissions. Since most of the N_2_O is emitted from the fertilized areas, experimental error is reduced by considering data obtained directly from chambers, which represent the fertilized bands. Additionally, emissions on a hectare basis can be easily calculated based on the emissions from the chambers. It is recommended to show both pieces of information in the studies. Researchers can then extrapolate the data for different conditions based on these findings. In the study, the cumulative N_2_O, CH_4_ and CO_2_ emissions were compared using orthogonal contrasts with SISVAR statistical software [22,23]. Given that the experiment utilized incomplete factorial designs, orthogonal contrasts were chosen as the preferred statistical analysis method. Orthogonal contrasts enable the comparison of different groups, facilitating a more efficient evaluation of specific effects.

As mentioned earlier, calculating the cumulative emissions for the entire system requires the use of transformation factors and the summation of background emissions. In the case of sugarcane, the fertilized bands with N fertilizer and CV vary in the literature, ranging from 16% (1600 m^2^ ha^−1^) to 20% (2000 m^2^ ha^−1^) of the total experimental area. The space between the fertilized bands (inter-row) ranges from 80% to 84% (8000 m^2^ ha^−1^ or 8400 m^2^ ha^−1^) [11,24]. In contrast, non-concentrated vinasse is applied across the entire area (100% or 10,000 m^2^ ha^−1^). In our study, we determined that the fertilized band accounted for 16% of the area, with the interrow comprising 84%. The decision was based on the majority of the papers found in the literature at that time [11,19,25,26].

The N_2_O—N emission factors (EF,%) must be calculated using emissions from the chambers, as the amounts of organic and inorganic N fertilizer placed in the chambers are known. But proper controls need to be implemented to account for the N_2_O emissions related to the treatment being evaluated. In the study, plots with no inorganic fertilizer and no vinasse, as well as plots with vinasse without fertilizer, were used to calculate the N_2_O—N EF. The EF is computed with the following Eq. (3):

(3) $$\text{EF}=\left\{\frac{N_{2}O_{\text{treat}}-N_{2}O_{\text{control}}\;}{N_{\text{applied}}\;\left(\text{fert}+\text{Vinasse}\right)}\right\}x\;100$$

where N_2_O_treat_ (mg N m^−2^) and N_2_O_control_ (mg N m^−2^) represent the cumulative emissions of the fertilized and unfertilized chambers, respectively, and N applied is the amount of N added to the chamber as ammonium nitrate and/or vinasse (CV or V).

Since there was a limitation related to the total number of chambers that could be installed in the field due to the high number of treatments, chambers with the treatments simulate the fertilized bands, and chambers from the control or the organic residue V (applied over the total area) simulate the inter-row area. For treatments with V, which were applied over the whole field, cumulative emissions on a hectare basis could also be calculated. Cumulative N_2_O—N emissions for V and mineral fertilizer were calculated by multiplying V plus N (V_a_+*N, V* + *N*, and *N*+V_p_) in mg N m^−2^ by 1.6 (1600 m2 ha^−1^) (16% of the total area) plus N_2_O emission from the area between rows (84%) with V, multiplying by 8.4 (8400 m^2^ ha^−1^). For all other treatments (fertilizer and CV) emissions for the cropping system can be calculated by multiplying the values in mg N m^−2^ by 1.6 since the inputs were applied just in the 0.25 m-wide band. EF is not affected by this calculation for all CV and N fertilizer treatments. For treatments containing V, the EF values are slightly different when computed on a field scale or per chamber area because the N contained in V applied in the interrow must be considered in the calculations.

However, if the researchers decide to calculate the EF on a field-scale basis (kg N_2_O—N ha^−1^), the EF can be calculated considering that the following equations [6,27]. The row emissions (kg N_2_O—N m^-2^) are multiplied by 1600 m^2^ ha^−1^. The calculation of the interrow emissions is different for V and for CV and N fertilizers, as V is applied in the whole area whereas the fertilizers and CV only in 16% of the field (row). For V, the interrow emissions are estimated by multiplying the chamber emissions of the V treatment, in kg N_2_O—N m^−2^, by 8400 m^2^ ha^−1^ [6]. In contrast, for the N fertilizers and CV, the chamber of control treatment emissions are used in the calculation [27]. The field-scale emissions are the sum of the band and the interrow emissions.

(4) $$EF_{V\;\text{treatments}}=\left\{\frac{\left[\left(N_{2}O_{\text{treat}}x1600\right)+\left(N_{2}O_{\text{Vinasse}}x8400\right)\right]-\left(N_{2}O_{\text{control}}x10000\right)\;}{N_{\text{applied}}\;\left(\text{fert}+\text{Vinasse}\right)}\right\}x\;100$$

(5) $$EF_{N\;\text{or}\;\text{CV}\;\text{treatments}}=\left\{\frac{\left[\left(N_{2}O_{\text{treat}}x2000\right)+\left(N_{2}O_{\text{control}}\;x8000\right)\right]-\left(N_{2}O_{\text{control}}x10000\right)\;}{N_{\text{applied}}\;\left(\text{fert}+\text{Vinasse}\right)}\right\}x\;100\;$$

where EF is the N_2_O—N emission factor (%), N_2_O_treat_, N_2_O_control_, and N_2_O_Vinasse_ are the cumulative emissions (kg m^−2^ of N_2_O—N) of the fertilized, unfertilized, and V treatment chambers, respectively, and N applied is the mass of N fertilizer added per hectare with N mineral fertilizer and N from vinasse (V and CV) (kg N ha^−1^).

## Soil sampling and chemical analysis: variables are related to GHG production

Soil nitrogen content, moisture, and temperature are particularly important variables related to GHG production. Therefore, it is recommended to sample soil in parallel to each gas sampling and also to have a meteorological station located close to the experimental area to obtain accurate climate-related data. Defining the soil sampling layer is also crucial since the objective is to comprehend how soil parameters influence GHG production. It is anticipated that these reactions occur on the soil surface. Based on this the selection of the 0–10 cm soil top layer was based on the presence of a more active microbial community in that layer. The choice of sample quantity, number of sub-samples, soil layer depth, and number of soil layers to be sampled is determined by the available budget and workforce to conduct the sampling, handle laboratory work, and manage the data. In the study example, in parallel to each gas sampling, air and soil temperatures were measured, and soil samples (six per block, 0–10 cm top layer) were collected for soil moisture analyses. In addition to the samples mentioned above, soil samples can be collected to measure pH, moisture content, water-filled pore space (WFPS), and concentrations of NO_3_^−^-N and NH_4_^+^-N (Fig. 1c). The timing of this type of sampling is crucial. This type of sampling should be conducted following expected high N_2_O production, focusing on intense soil sampling immediately after fertilizer application, especially after rain events. The selection of variables to analyze should align with the research question of the experiments. In our study, we prioritized the analysis of mineral N in the soil, as well as moisture content, as these are key factors influencing N_2_O emissions. However, depending on the specific research question, a range of other analyses can be conducted, including assessments of labile organic C, available K, P, and more. In this particular study, total N and C likely did not change in the soil because vinasses were applied for the first time in the area. Therefore, these variables were not analyzed. However, in a long-term experiment, both factors can influence N_2_O emissions significantly.

The type of analysis will determine the sampling strategies and how the samples are stored during transport and in the laboratory. Soil samples intended for mineral N analysis must be kept fresh. It is recommended to transport them in containers with ice, especially in warm regions, to reduce microbial activity during transport. If possible, the fresh samples should be analyzed in the laboratory immediately. If the same samples are intended to be used for other analyses, such as microbiology analyses, different temperatures are required. For example, for DNA extraction, they should be kept at −20 °C. However, for RNA extraction, the samples should be stored at −80 °C to maintain their quality and stop the microbial activity allowing for a snapshot of that particular moment when the soil sampling was done. If the goal is to isolate microorganisms, the samples must be stored under specific conditions suitable for each microbe being studied.

In the study, soil samples for chemical analyses were stored at −20 °C, and a portion (30 g) was kept at −80 °C for molecular analyses. During our example study, the soil was sampled 41, 23, and 19 different days were for R1, D1, and R2 experiments, respectively. The reduced number of soil samples in the D2 and R2 experiments was based on the knowledge acquired about N_2_O emissions when both vinasses were applied to the soil. These insights guided the sampling strategy for the subsequent experiments. The rationale behind this sampling approach is that immediately after the application of vinasse and mineral N, high N_2_O fluxes are expected. Similar emissions occur after rain events if fertilizer is present in the system. If the objective is to determine the pathways related to N_2_O production, soil samples need to be collected right after these events. It is challenging and unnecessary to collect soil samples during every GHG sampling throughout the entire experiment (one year). Researchers should be aware that the primary N_2_O emission peaks occur immediately after N fertilization. Therefore, GHG and soil sampling must be intensive during this first period. A single day of sampling (high gas flux) can significantly impact the cumulative emissions data.

In our study, the soil sampling started before the application of inorganic fertilizer and vinasse, with the final soil samples collected when mineral N levels in fertilized treatments were comparable to those in the control treatment. However, in the case of D1, due to research inquiries about soil microbiome dynamics, the last soil sampling took place just before harvesting. The extensive soil sampling in the experiments was necessary for subsequent analyses, particularly for determining the processes responsible for N_2_O production, as will be described in the following section. For research studies that are not explicitly studying the pathways related to N_2_O emission, a weekly soil sampling frequency is generally sufficient to correlate chemical factors, such as mineral N, with N_2_O emissions. This frequency strikes a balance between obtaining meaningful data and optimizing resources.

The methods selected to determine the variables can vary based on the resources and equipment available. In the study, soil moisture was determined gravimetrically by drying the soil at 105 °C for 24 h, and all results were expressed per gram of dry soil. The water-filled pore space (WFPS) was calculated considering soil moisture, soil bulk density and porosity determined at the beginning of the experiment [28]. The mineral N was extracted with 1 M KCl in 1:10 soil-to-solution ratio and the concentrations of NH_4_^+^ [29] and NO_3_^−^ [30] in the filtered extract were determined colorimetrically by flow injection analysis (FIAlab-2500 System). The air and soil temperatures were measured using a digital soil thermometer. Climatic data were obtained from a meteorological station located approximately 500 m from the experimented area.

## Plant productivity and quality

The sampling methods and plant parts to be analyzed vary depending on the plant species. These can include grains, leaves, stalks, roots, and more. In our specific case, we determined sugarcane productivity using the biometric method (Fig. 1b). The stalk yield was estimated by counting and weighing the stalks along a 2 m stretch of two sugarcane rows in each plot (totalling 6 m^2^ ), which were randomly collected. If a machine is available, it is also possible to weigh the entire stalk production of each plot. Additionally, samples of sugarcane stalks, tops, and dry leaves were collected from the same area to determine dry matter and nutrient accumulation (results not published). These samples were weighed, chopped, and subsamples were then oven-dried at 40  °C until a constant weight was achieved. The dried stalks, tops, and dry leaves samples were ground to a fine powder before measuring macro (N, P, K, Ca, Mg, and S) and micronutrients (Fe, Cu, Zn, Mn, and B) using the method described by Bataglia et al. [8], a method commonly used in São Paulo state of Brazil.

## DNA extraction

The production of N_2_O emitted from the soil is primarily carried out by microbes [[31], [32], [33], [34]]. Different microbes possess unique genetic mechanisms responsible for this production. Therefore, elucidating the primary microbial pathways related to N_2_O emissions from the soil is necessary. Identifying the microbes responsible for the majority of N_2_O production from soils when organic and inorganic fertilizers are applied will enable researchers to develop targeted strategies. It's worth mentioning that N_2_O emissions from N fertilizers are associated with avoided emissions. While N_2_O is naturally produced, the current emission rates far exceed natural levels. Based on this premise, experiments aimed at measuring GHG emissions can provide valuable insights into the pathways associated with these emissions. In this particular study example, two field experiments were conducted to collect soil samples to identify the key microbes correlated with N_2_O production. As mentioned earlier, a substantial number of soil samples were collected. In experiment R1, 41 soil samplings were carried out, while in experiment R2, 23 soil samplings were conducted. The intervals between collection days varied according to the timing of fertilization and rain events. However, due to budget constraints, only 6 soil samplings from each experiment (12 timepoints in total) were selected for further analysis.

To assess microbes correlated with N_2_O production, it is essential to analyze days with both high and low emissions. Quantitative real-time PCR analyses (qPCR), a molecular biology technique, to quantify the number of copies of each of the tested genes was employed. For this purpose, soil samples 12 timepoints (a total of 10 treatments with 3 replicates) across all treatments in the R1 and D1 experiments and subjected to DNA extraction. The soil sampling chosen for DNA extraction was performed at 1, 3, 7, 22, 24, and 54 days after inorganic N application in R1 and −30, 1, 11, 19, 45, and 52 days after inorganic N application in D1. But beyond assessing the pathways related to N_2_O production, the approach used here can be used to investigate the broader impact caused by the application of organic and inorganic fertilization on the total microbial community. By using the same DNA samples, it is possible to assess a variety of agronomic as well as ecological research questions. The changes in the soil microbiome were evaluated in experiment D1, and soil samples (4 treatments x 3 replicates) were collected at 1, 3, 8, 31, 36, 42, 50, 76, 113, 183, and 389 days after the beginning of the experiment. The selection of the number of treatments, replicates, and time points was based on the cost of each analysis. As mentioned earlier, the cost of analyses can influence the number of samples and replicates feasible for each study. Molecular analyses, in particular, can be expensive when compared with others, serving as a limiting factor. In this study, the experiments were structured in a randomized block design with ten treatments and four blocks (40 plots). However, for molecular analyses, two experiments and three blocks were chosen for soil DNA extraction. Therefore, it was decided to work with fewer than 400 DNA samples (360 DNA samples from soil and 21 DNA samples from vinasses).

A diverse range of kits for isolating DNA is available, and in this study, the MoBio PowerSoil DNA Isolation Kit (MoBio, Solana Beach, CA, USA) was employed. Total DNA was extracted from 0.25 g of each soil sample following the manufacturer's instructions. All DNA samples were stored at −20  °C until they were used in downstream analyses. Three replicates of each vinasse batch were also used for DNA extraction. To extract the DNA from an amendment such as vinasse, two 50-mL aliquots of each vinasse replicate were centrifuged at 10,621 g for 10 min using a benchtop centrifuge (Sigma 2–16P) to separate the cells from the liquid. The resulting pellets were then combined for further analysis. This can applied also to manure, slurry, compost, etc. Total DNA was extracted from the pellets using the MoBio PowerSoil DNA Isolation Kit (MoBio, Solana Beach, CA, USA) following the manufacturer's instructions. The concentration and quality of both soil and vinasse DNA samples were assessed spectrophotometrically (NanoDrop 1000, Thermo Scientific, Waltham, MA, USA), fluorometrically (Qubit 2.0 Fluorometer, Life Technologies, Carlsbad, CA, USA), and via agarose gel electrophoresis (Tris-acetate-EDTA (TAE) buffer, 1% w/v).

As mentioned before, a wide range of questions can arise during the projects. The DNA from the vinasse was also utilized to characterize the microbial assemblage of vinasse. The objective was to determine the gene potential of microbes contributing to negative environmental effects during fertirigation and/or hindering bioethanol fermentation. The methodology used and the main results are described in Cassman et al. [35].

## Quantitative real-time PCR analyses

### Reactions

A wide array of microbial genes is associated with N_2_O production and consumption. Consequently, researchers must carefully select the most significant genes for analysis. Adequate funding and skilled technicians with expertise in this type of analysis are essential for accurate and meaningful results. In the study, the abundances of ribosomal RNA genes were used as proxy for total bacteria (16S rRNA) and total fungi (18S rRNA). Additionally, functional genes, including *amoA* (bacterial and archaeal), *nirS* (bacterial), *nirK* (bacterial and fungal), and *nosZ* (bacterial), which encode proteins involved in nitrification and denitrification processes, were quantified using quantitative real-time PCR (qPCR). Soil DNA and vinasse samples were analyzed with two technical replicates to ensure the reliability of the results. qPCR was performed in a 96-well plate (Bio-Rad) using the CFX96 TouchTM Real-Time PCR Detection System (Bio-Rad). qPCRs were performed in a total volume of 12 µl containing 4 µl of DNA, except fungal nirK, which was amplified in a total volume of 10 µl containing 1 µl of undiluted DNA due to the low number of gene copies. The reaction descriptions, primer combinations, and thermal cycler conditions for each gene amplification are listed in Table 2. Data were acquired at 72 °C, and melting curve analysis was performed to confirm specificity. Amplicon sizes were confirmed on 1% (w/v) agarose gels. It was used plasmids that contained the gene of intereste cloned, then they were used to construct standard curves [26]. Standard curves were performed 10 times using serial dilutions from 10 to 10^−8^. The reaction efficiency varied from 80% to 105%, and the R^2^ values ranged from 0.94 to 0.99. The methodologies used were calibrated to the soil and to the Bio-Rad qPCR machine used, each researcher must test the efficiency of the qPCR reactions and adapt them to the specific conditions. Researchers need to take into account that the qPCR technique was developed for amplicons with small sizes, up to 200 bp. Consequently, primers amplifying larger amplicons, such as the gene *amoA*, will exhibit lower reaction efficiency compared to 16S rRNA and bacterial *nirK*. Researchers should be prepared to adapt qPCR conditions to their specific requirements and verify the efficiency of their reactions accordingly.

**Table 2:** Primers and thermocycler conditions used in gene abundance analysis by qPCR.

Target gene	Primers	Primer Sequence	Amplification size (bp)	Reaction	Cycling conditions	Reference
				**12 µL of reaction**		
AOA *amoA*	Arch-amoAF Arch-amoAR	5′-STAATGGTCTGGCTTAGACG-3′ 5′-GCGGCCATCCATCTGTATGT-3′	635	6 µL of Sybrgreen Bioline SensiFAST SYBR non-rox mix, 0.125 µL of each primer (10 pmol), 1.75 µL of BSA and 4 µL of DNA (40 ng).	95 °C-5 min.; 40 × 95 °C-10 s, 64 °C-10 s, 72 °C-20s	Francis et al. [36]
AOB *amoA*	amoA1F amoA2R	5′-GGGGTTTCTACTGGTGGT-3′ 5′-CCCCTCKGSAAAGCCTTCTTC-3′	491	6 µL of Sybrgreen Bioline SensiFAST SYBR non-rox mix, 0.125 µL of each primer (10 pmol) and 4 µL of DNA (40 ng).	95 °C-10 min.; 40 × 95 °C-10 s, 65 °C-25 s,	Rotthauwe et al. [37]
*nosZ*	nosZ2F nosZ2R	5′-CGCRACGGCAASAAGGTSMSSGT-3′ 5′-CAKRTGCAKSGCRTGGCAGAA-3′	267	6 µL of Sybrgreen Bioline SensiFAST SYBR non-rox mix, 0.250 µL of each primer (10 pmol), 1.20 µL of BSA and 4 µL of DNA (5 ng).	95 °C-5 min.; 40 × 95 °C-10 s, 64 °C-10 s, 72 °C-20s	Henry et al. [38]
*nirK*	NirK876 NirK1040	5′-ATYGGCGGVAYGGCGA-3′ 5′-GCCTCGATCAGRTTRTGGTT-3′	165	6 µL of Sybrgreen Bioline SensiFAST SYBR non-rox mix, 0.250 µL of each primer (10 pmol), 1.50 µL of BSA and 4 µL of DNA (5 ng).	95 °C-5 min.; 40 × 95 °C-15 s, 62 °C-15 s, 72 °C-20 s	Henry et al. [39]
*nirS*	nirScd3aF nirSR3cd	5′-GTSAACGTSAAGGARACSGG-3′ 5′-GASTTCGGRTGSGTCTTGA-3′	425	6 µL of Sybrgreen Bioline SensiFAST SYBR non-rox mix, 0.250 µL of each primer (10 pmol), 1.20 µL of BSA and 4 µL of DNA (5 ng).	95 °C-5 min.; 40 × 95 °C-10 s, 63 °C-10 s, 72 °C-20s	Throbäck et al. [40]
16S rRNA	Eub338 Eub518	5′-ACTCCTACGGGAGGCAGCAG-3′ 5′-ATTACCGCGGCTGCTGG-3′	200	6 µL of Sybrgreen iQ™ SYBR® Green Supermix (Bio-Rad), 0.125 µL of each primer (10 pmol), 0.30 µL of BSA and 4 µL of DNA (5 ng).	95 °C-3 min.; 40 × 95 °C-30 s, 59 °C-35 s, 72 °C-20s	Fierer et al. [41]
18S rRNA	FF390 FFR1	5′-CGATAACGAACGAGACCT-3′ 5′-AICCATTCAATCGGTAIT-3′	390	6 µL of Sybrgreen iQ™ SYBR® Green Supermix (Bio-Rad), 0.250 µL of each primer (10 pmol), 0.30 µL of BSA and 4 µL of DNA (5 ng).	95 °C-3 min.; 40 × 95 °C-30 s, 52 °C-45 s, 72 °C-50 s	Vainio and Hantula [42]
				**10 µL of reaction**		
*nirK* fungi	fnirK2F fnirK1R	5′-GTYCAYATYGCYAACGGSATGTACGG-3′ 5′-GCRTGRTCNACMAGNGTRCGTCCC-3′	468	5 µL of Sybrgreen PowerUp™ SYBR® Green Master Mix (ThermoFisher), 0.250 µL of each primer (10 pmol) and 1 µL of DNA (undiluted).	50 °C-2 min; 95 °C-2 min; 45 × 95 °C-15 s, 52 °C-30 s, 72 °C-60 s	Long et al. [43]

The interpretation of *nirK*-Fungi gene results is subject to methodological limitations. While the limitation in connecting the fungal role to N_2_O production has decreased with recently designed fungal *nirK* and *P450nor* PCR primers [[43], [44], [45], [46], [47]], issues with primer specificity persist. Despite the positive correlation of qPCR nirK-fungi, we acknowledge that the primers for fungal denitrifiers are degenerate and could also amplify bacterial-*nirK* [48,49]. To address this limitation, correcting the number of *nirK* copies by fungal denitrifier relative abundance for each sample is possible [35,48]. However, researchers must possess the *nirK*-fungi sequence data to employ this methodology. Using this approach, we obtained similar results; the N_2_O emission was correlated with both raw and treated *nirK*-fungi data [48].

Analyzing gene abundance in theory might seem like a straightforward technique, but in practice, defining the protocols requires a significant amount of time and energy. Having a qualified expertise in molecular biology is of fundamental importance in this process. The availability of funds and well-defined protocols serve as the bottleneck for conducting this type of analysis efficiently and accurately. Proper investment in both skilled personnel and robust protocols is crucial for successful gene abundance studies.

### Statistical analysis

Dealing with data that does not adhere to the assumptions of normality poses significant challenges in scientific research. Non-normally distributed data can complicate statistical analyses, making it difficult to apply conventional parametric tests that assume a normal distribution. Researchers often encounter skewed or non-normally distributed data, especially in fields where complex and multifaceted variables are studied, such as GHG emissions and microbial abundance data. One of the major issues with non-normally distributed data is the limited availability of appropriate statistical methods. Traditional parametric tests, such as *t*-tests or ANOVA, rely on the assumption of normality and may provide inaccurate or biased results when applied to non-normally distributed data. Correlation analyses, particularly Pearson correlation, assume normality and are sensitive to deviations from this assumption.

Addressing this challenge requires researchers to explore alternative methods designed for non-normally distributed data. Non-parametric tests, like Spearman's rank correlation. Spearman's correlation do not require the data to follow a normal distribution, making them suitable for correlating variables when normality assumptions are violated. Additionally, researchers can employ data transformation techniques to normalize the data, making it suitable for traditional parametric tests. Common transformations include logarithmic, square root, or Box-Cox transformations, which can help approximate normality in the data. Despite these strategies, it is crucial for researchers to approach the analysis with caution, ensuring that the chosen methods align with the nature of the data and the research question. Exploratory data analysis and careful consideration of the data's distributional properties are essential steps in determining the appropriate statistical approach. Collaboration with statisticians or data scientists can also be valuable, as they can offer expert guidance on selecting the most suitable methods for analyzing non-normally distributed data and correlating variables accurately.

In the study, gene abundance was assessed in gene copies per gram of dry soil. Spearman correlation analysis (Systat Software, 2014) was employed to calculate the correlations between microbial gene abundance and N_2_O flux. Furthermore, to assess the relative impacts of functional genes, treatments, and climatic factors on N_2_O emissions, a general linear model was applied for both seasons. The general linear model incorporated the lasso penalty and was computed using cyclical coordinate descent along a regularization path, following the approach outlined by Friedman et al. [50]. Log10 transformation was applied to N_2_O and gene abundance data (including N_2_O, 16S rRNA, and 18S rRNA, archaeal and bacterial *amoA*, fungal and bacterial *nirK*, bacterial *nirS*, and *nosZ*), as well as standardized soil factor variables. The lasso penalty, a regression method performing variable selection and shrinkage, was employed [51]. Cross-validation criteria with the “one standard error” rule were adopted, checking the lambda value that minimized the mean square error and selecting the largest value of lambda within one standard error of the minimum, to determine the most appropriate model [27]. This criterion enabled the selection of a model minimizing both the selected variables and square error. Treatments were incorporated as dummy variables, and the analysis was conducted using the “GLMNET” package in the R environment [50].

## Soil microbiome analyses

Sequencing DNA data derived from soil samples is crucial when organic and inorganic fertilizers are applied to the soil as it provides valuable insights into the dynamic interactions between bacteria, fungi, and fertilizers. Understanding the genetic composition of soil microbial communities is essential for unravelling the intricate processes related to nutrient cycling, organic matter decomposition, and GHG emissions. DNA sequencing enables the identification of specific bacterial and fungal species present in the soil, allowing researchers to assess their roles in nutrient transformations and emissions such as N_2_O. Indeed, studying the genetic diversity and abundance of microorganisms in response to fertilizer application is invaluable for comprehending the complexity of soil microbiomes. This understanding forms the foundation for developing sustainable agricultural practices that efficiently utilize nutrients, mitigate environmental impacts, and improve overall soil health. In the current context, where the sustainability of agricultural systems is paramount, such research plays a pivotal role in ensuring the long-term health and productivity of our ecosystems.

A crucial aspect to emphasize is the significance of conducting time-series analyses. Time-series analyses are important to understanding the dynamics of soil bacterial and fungal communities in response to the application of organic and mineral fertilizers. By observing changes over time, researchers can identify patterns, fluctuations, and trends in microbial populations. This temporal perspective allows scientists to pinpoint specific moments of impact, discern the duration of these effects, and comprehend the overall resilience and adaptability of the microbial communities. Time-series analyses provide valuable insights into the complex interplay between fertilization, microbial populations, and environmental factors. This understanding is essential for implementing precise and sustainable agricultural practices, ensuring the health and productivity of soil ecosystems in the long run.

In the study, the total soil bacterial and fungal communities were evaluated over a period of 389 days [3,4]. Analyzing the changes in these communities over time was essential to comprehend the impact of the organic residue vinasse on the microbes. This extended timeframe allowed researchers to observe the gradual shifts, adaptations, and responses within the bacterial and fungal populations. By conducting analyses over nearly a year, the study gained a comprehensive understanding of the long-term effects of vinasse application on soil microbial dynamics, providing valuable insights into sustainable agricultural practices and environmental impact mitigation. The number of soil samples and the time period between sampling days were initially chosen based on CO_2_ and N_2_O emissions, as a significant impact was expected immediately after the application of organic and mineral fertilizers in the soil. CO_2_ emitted from the soils is related to microbial and root respiration; higher CO_2_ emissions are usually correlated with higher microbial activity. Later on, a more stable community was anticipated. Additionally, the available budget for sequencing the samples also influenced the sampling frequency.

### 16S rRNA gene amplification, sequencing and data analysis

The 16S ribosomal RNA (rRNA) gene is a key component of the bacterial genome, and it is important for the identification and classification of bacterial species. It contains both conserved regions, which are similar across all bacteria, and variable regions, which exhibit variations that are unique to different bacterial taxa. These variable regions are crucial for distinguishing between bacterial species or genera. Different variable regions of the 16S rRNA gene can be targeted for sequencing, and the choice of region depends on the research goals and the level of taxonomic resolution required. In the study, the variable V4 region of bacterial 16S rRNA gene was utilized as targets for the analyses of Illumina 16S rRNA gene sequencing using the universal bacterial primers 515F (5′-GTGCCAGC MGCCGCGGTAA-3′) and 806R (5′- GGACTACHVGGGTWTCTAAT-3′) resulting in amplicons of ∼300–350 bp [52,53]. The primers already contain barcodes and sequencing adapters. The reactions were made in triplicate, they were performed in a total volume 25 µl containing 18.3 µl of H_2_O, 2.5 µl of 10X PCR-buffer + magnesium, 2.5 µl of dNTPs (2 mM), 0.20 µl of fast startExp-Polymerase (5 U µL^−1^), 0.25 µl of each primer (10 pmol µl^−1^) (forward and reverse) and 1 µl of DNA template in a thermocycler (Bio-Rad, CA, USA) with the following conditions: initial denaturation for 5 min at 95  °C, followed by 35 cycles of 30 s at 95  °C, 30 s at 53  °C and 60 s at 72  °C and a final extension for 10 min at 72  °C. Each sample was purified with Agencourt AMPure XP beads (Beckman Colter, Berea, CA, USA), and the library quality was assessed on a Qubit 2.0 Fluorometer (Thermo Scientific) and an Agilent Fragment Analyzer system. The samples were pooled based on the concentration of 20 ng ul^-1^ of DNA for each sample. Dual-index and Illumina sequencing adapters were attached to the V4 amplicons. The mixture of PCR products was subjected to paired-end sequencing using a MiSeq Reagent Kit v3 on the Illumina MiSeq Sequencing platform on the Illumina MiSeq System at BGI Genomics, China.

The composition of bacterial communities of the soil samples were analyzed based on the raw sequencing data obtained from the Illumina MiSeq platform. Sequencing reads were sorted into each sample according to their barcode sequences. The sequences were quality filtered with a Phred score of at least 25, expected errors of 0.5 and length of 200 bp. Paired-end sequences were overlapped to assemble amplicon regions with PANDASeq with a minimum overlap of 50 bp [54]. Operational taxonomic units (OTUs) were clustered at 97% sequence similarity using the UPARSE pipeline after discarding the singletons and replicates across the data set [55]. Sequences were screened for chimeras using de novo mode in UCHIME [55] and the resulting chimeric sequences were removed. A representative sequence was chosen from the most abundant sequence for each OTU, and taxonomy was assigned using BLAST against the Greengenes 13.5 database with a confidence threshold of 80%.

Researchers have recently shifted from using OTUs to Amplicon Sequence Variants (ASVs) for analyzing microbial sequence data. During our study, the ASV concept was not widely adopted and not commonly found in the literature. The growing preference for ASVs over OTUs in microbial community analyses stems from the desire to achieve the highest taxonomic resolution possible, reflecting species-level taxonomy. ASVs offer superior resolution compared to OTUs, enabling the differentiation of closely related sequences and allowing researchers to identify subtle differences within microbial communities. Unlike OTUs, ASVs eliminate the need for a clustering step since each ASV represents a unique biological sequence. However, using ASVs increases the risk of splitting a single genome into separate clusters [56]. Although similar risks exist when employing narrow thresholds to identify OTUs, these concerns are less significant than artificially splitting a genome into distinct ASVs. Regardless of the chosen approach, whether OTUs or ASVs, analyses conducted on an OTU dataset, including those proposed in this study, can be applied to ASV datasets seamlessly.

### ITS amplicon amplification, sequencing and data analysis

The Internal Transcribed Spacer (ITS) region is a genetic marker commonly used in the study of fungal diversity and taxonomy. Similar to the 16S rRNA gene for bacteria, the ITS region is used to characterize and identify different fungal species based on genetic variations. The ITS region comprises two distinct subregions: ITS1 and ITS2, separated by the 5.8S rRNA gene. These regions are found within the ribosomal RNA gene cluster in the fungal genome. The fungal ribosomal internal transcribed spacers (region ITS2) region was utilized as targets for the analyses of Illumina ITS gene sequencing. The ITS2 primers ITS9F [57] and ITS4R [58] primers (5′GAACGCAGCRAAIIGYGA-3′ and 5′-TCCTCCGCTTATTGATATGC-3′) with barcodes were used for PCRs amplifications. The reaction was conducted in 25 µl containing 15.6 µl of H2O, 2.5 µl of 10X PCR-buffer + magnesium, 2.5 µl of dNTPs (2 mM), 0.15 µl of fast startExp-Polymerase (5 U/µL), 1 µl of MgCl_2_ (25 mM), 1.25 µl of BSA, 0.5 µl of each primer (forward and reverse) and 1 µl of DNA template in a thermocycler (Bio-Rad, CA, USA) with the following conditions: initial denaturation for 5 min at 95  °C, followed by 40 cycles of 45 s at 95  °C, 60 s at 54  °C and 90 s at 72  °C and a final extension for 10 min at 72  °C. Each sample was purified with Agencourt AMPure XP beads (Beckman Colter, Berea, CA, USA), and the library quality was assessed on a Qubit 2.0 Fluorometer (Thermo Scientific) and an Agilent Fragment Analyzer system. The samples were pooled based on the concentration of 20 ng/ul of DNA for each sample. Finally, the mixture of PCR products was subjected to paired-end sequencing using a MiSeq Reagent Kit v3 on the Illumina MiSeq Sequencing platform at BGI Genomics, China.

The composition of fungal community of the soil samples were analyzed based on the raw sequencing data obtained from the Illumina MiSeq platform. Sequencing reads were sorted into each sample according to their barcode sequences. The sequences were filtered according to quality and length, adapter sequence trimming and PhiX contaminant removal with BBDuk2 from the BBMap tool suite [59]. Paired-end sequences were overlapped using VSEARCH [60]. Operational taxonomic units (OTUs) were clustered at 97% sequence similarity with the usearch_global method implemented in VSEARCH after discarding the singletons across the data set [55]. Sequences were screened for chimeras using de novo mode in UCHIME [55] and the resulting chimeric sequences were removed. A representative sequence was chosen from the most abundant sequence for each OTU, and taxonomy was assigned using RDP Classifier against the Unite database 7.2 as a reference with a confidence threshold of 80%.

### Bioinformatics and data analysis

Analyzing genomic data, particularly sequencing data from genes like 16S rRNA and 18S rRNA, poses significant challenges and holds immense importance in the field of biological research. These challenges stem from the large volume and complexity of the data, requiring advanced computational tools to process and decipher meaningful patterns. Biological variability and the dynamic nature of microbial communities add layers of complexity, requiring sophisticated analytical techniques to distinguish between closely related species and capture temporal and spatial dynamics. Selecting the appropriate statistical analyses for each study poses a significant challenge, especially when evaluating the impact of organic and inorganic fertilizer addition. To navigate this complexity, researchers can examine the methodologies employed in previous studies with similar research objectives. By studying the analyses used in these studies, researchers can gain valuable insights into effective statistical approaches. Understanding the nuances of these methodologies allows scientists to make informed decisions about which statistical tools are best suited for their own research, enabling them to follow the analyses that have been proven effective in similar contexts. Based on this, follow the statistical analyses that were used in the study.

Downstream analyses were done with an OTU table normalized with Hellinger's transformation for bacterial and fungal communities, separately. Sampling effort can be evaluated differently. For bacteria, sampling effort was estimated by Good's coverage [61] while for fungi, rarefaction curves from non-rarefied data using the sequence sample size and number of different OTUs were calculated in RStudio version 1.0.136 running R version 3.3.1 using the phyloseq [62] and ranacapa [63] packages. The good's coverage estimates how representative samples of bacterial community are. Values greater than 99% indicate high coverage of sampling community and that the sequencing depth used provides an accurate view of microbial community diversity. In the same line, rarefaction allows the calculation of species richness for a given number of reads randomly drawn from the data, for each sample, based on the construction of so-called rarefaction curves. This curve is a plot of the number of species as a function of the number of samples. Rarefaction curves generally grow rapidly at first, as the most common species are found, but the curves plateau as only the rarest species remain to be sampled.

For both bacteria and fungi, estimates of alpha diversity were calculated in QIIME 1.9 [52]. Alpha diversity metrics summarize the structure of an ecological community with respect to its richness (number of taxonomic groups), evenness (distribution of abundances of the groups), or both. These estimates included the observed Chao1, Simpson and Shannon diversity indices [64]. Different α-diversity metrics reflect different views on the true diversity and they perform differently. For example, Richness (also known as Observed or Chao1), Shannon [65,66] and Simpson [67] indices are non-phylogenetic metrics (i.e., based solely on abundance information) which weight relatively rare, mid-abundant and abundant species, respectively. As alpha diversity measures are sensitive to differences in sampling effort, estimates were calculated based on rarefied data sets. Rarefaction is a method that adjusts for differences in library sizes across samples to aid comparisons of alpha diversity.

Multivariate dispersion analysis was performed to test the differences in variances among the treatments using the PERMDISP2 procedure in PRIMER v7 software. Permutational multivariate analysis of variance (PERMANOVA) was used to assess statistical differences in each community composition among treatments. Factors in the PERMANOVA were indicated as well as their interactions using the ‘vegan’ package with 10,000 permutations [68]. Ordination of the 16S rRNA and ITS2 region sequences were investigated using principal coordinate analysis (PCoA) implemented in QIIME 1.9 [52] for bacteria and using a discriminant analysis of principal components (DAPC) implemented in the ‘adegenet’ R package [69] for fungi. However, the analyses could be used for both communities, it is a matter of choice. PCoA is a method to explore and visualize similarities or dissimilarities of data. It starts with a similarity matrix or dissimilarity matrix and assigns for each item a location in a low-dimensional space. PCOA tries to find the main axes through a matrix and calculates a series of eigenvalues and eigenvectors. In the other side, the samples in DAPC are partitioned into a between-group and within- group component, in an effort to maximize discrimination between groups. In DAPC, data is first transformed using a principal components analysis (PCA) and subsequently, clusters are identified using discriminant analysis (DA).

Multivariate regression tree (MRT) analyses (Fig. 1e) based on Bray–Curtis dissimilarities of the bacterial and fungal communities using the function “mvpart” was also used to explore the dynamics of communities over time for each treatment [70]. For the analysis, the data were log-transformed, and the resulting tree was plotted after 500 cross-validations [21] to prevent overfitting. Next, the PCoA of the MRT was plotted with the function “rpart.pca” from the “mvpart” package [71]. Multivariate regression tree (MRT) analysis was used to estimate the impact of time on the bacteria and fungal communities’ structure independently for each treatment. In the PCoA of the MRT different leaves (large colored circles) were defined based on bacterial and fungal abundance and composition. The community composition within leaves is represented in a principal coordinate analysis (PCoA) plot, where small points represent individual samples and large points represent the group mean (within the leaf). The grey barplot in the background of each graphic indicates families (level that we used) whose differential abundance explains the variation in the PCoA plot.

Individual indicator microbial families associated with treatments and days were identified using ‘linear discriminant analysis effect size (LEfSe) in Microbiome Analyst [72], a web-based tool, to identify the families that were most enriched in the soil [73]. Based on the normalized relative abundance matrix, the LEfSe method uses the Kruskal–Wallis rank-sum test to detect features with significantly different abundances between the assigned taxa and performs linear discriminant analysis (LDA) to estimate the effect size of each feature. A significance level of α≤ 0.05 was used for all biomarkers evaluated in this study.

To display co-trends between the fungal and bacterial communities, data were analyzed using co-inertia analysis computed with the ‘ade4’ package in R [74]. This method calculates the covariance of two taxa tables and plots a common ordination space of co-inertia axes in principal component analyses (PCA) aiming to maximize the concordance between the data sets. To evaluate the invasiveness of the microbial community to the soil, the bacterial and fungal OTUs were evaluated according to their presence in vinasse residue and tracing back their survival and persistence on the collected soil samples. Redundancy analysis (RDA) also can be used to identify significant covariates among microbial communities, physicochemical characteristics and aboveground as greenhouse gas fluxes were determined by redundancy analysis (RDA) with the Hellinger transform OTU table using CANOCO 5 (Biometris, Wageningen, The Netherlands).

### Papers and thesis derived from field experiments

In this section, we highlight the papers that have been published based on the experiments conducted. The research outcomes from our field studies have contributed significantly to the scientific community. These publications showcase the diverse range of topics explored and the impactful findings derived from our experiments. Each paper represents a meticulous analysis of specific aspects of our research, offering in-depth insights into various agronomic practices, environmental impacts, and innovative methodologies. The following list provides a summary of the key papers, illuminating the depth and breadth of knowledge generated through these experiments. These publications stand testament to the collaborative efforts, expertise, and dedication of the research team involved in this endeavor.

Galdos, M.V., Soares, J.R., Lourenço, K.S., Harris, P., Zeri, M., Cunha-Zeri, G., Vargas, V.P., Degaspari, I.A.M., Cantarella, H., 2023. Multi-experiment assessment of soil nitrous oxide emissions in sugarcane. Nutrient Cycling in Agroecosystems. https://doi.org/10.1007/s10705–023–10,321-w

Lourenço, K.S., Costa, O.Y.d.A., Cantarella, H., Kuramae, E.E., 2022. Ammonia-oxidizing bacteria and fungal denitrifier diversity are associated with N2O production in tropical soils. Soil Biology and Biochemistry 166. https://doi.org/10.1016/j.soilbio.2022.108563

Carvalho, J.L.N., Oliveira, B.G., Cantarella, H., Chagas, M.F., Gonzaga, L.C., Lourenço, K.S., Bordonal, R.O., Bonomi, A., 2021. Implications of regional N2O–N emission factors on sugarcane ethanol emissions and granted decarbonization certificates. Renewable and Sustainable Energy Reviews 149, 111,423. https://doi.org/10.1016/j.rser.2021.111423

Lourenço, K.S., Suleiman, A.K.A., Pijl, A., Cantarella, H., Kuramae, E.E., 2020. Dynamics and resilience of soil mycobiome under multiple organic and inorganic pulse disturbances. Science of the Total Environment, 139,173. https://doi.org/10.1016/j.scitotenv.2020.139173

Lourenço, K.S., Rossetto, R., Vitti, A.C., Montezano, Z.F., Soares, J.R., de Melo Sousa, R., do Carmo, J.B., Kuramae, E.E., Cantarella, H., 2019. Strategies to mitigate the nitrous oxide emissions from nitrogen fertilizer applied with organic fertilizers in sugarcane. Science of the Total Environment 650, 1476–1486. https://doi.org/10.1016/j.scitotenv.2018.09.037

Cipriano, M.A.P., Suleiman, A.K.A., da Silveira, A.P.D., do Carmo, J.B., Kuramae, E.E., 2019. Bacterial community composition and diversity of two different forms of an organic residue of bioenergy crop. PeerJ 7, e6768-e6768. https://doi.org/10.7717/peerj.6768

Lourenço, K.S., Suleiman, A.K.A., Pijl, A., van Veen, J.A., Cantarella, H., Kuramae, E.E., 2018. Resilience of the resident soil microbiome to organic and inorganic amendment disturbances and to temporary bacterial invasion. Microbiome 6. https://doi.org/10.1186/s40168–018–0525–1

Lourenço, K.S., Dimitrov, M.R., Pijl, A., Soares, J.R., do Carmo, J.B., van Veen, J.A., Cantarella, H., Kuramae, E.E., 2018. Dominance of bacterial ammonium oxidizers and fungal denitrifiers in the complex nitrogen cycle pathways related to nitrous oxide emission. GCB Bioenergy 10, 645–660. https://doi.org/10.1111/gcbb.12519

Lourenço, K.S., Cassman, N.A., Pijl, A.S., van Veen, J.A., Cantarella, H., Kuramae, E.E., 2018. Nitrosospira sp. govern nitrous oxide emissions in a tropical soil amended with residues of bioenergy crop. Frontiers in Microbiology 9. https://doi.org/10.3389/fmicb.2018.00674

Cassman, N.A., Lourenço, K.S., do Carmo, J.B., Cantarella, H., Kuramae, E.E., 2018. Genome-resolved metagenomics of sugarcane vinasse bacteria. Biotechnology for Biofuels 11, 48. https://doi.org/10.1186/s13068–018–1036–9

Lourenço, K.S., 2018. Linking soil microbial community dynamics to N2O emission after bioenergy residue amendments, Institute of Biology Leiden (IBL), Science. Leiden University, p. 173.

Lourenço, K.S., 2018. Strategies to mitigate the nitrous oxide emissions from inorganic fertilizers plus vinasse, Gestão de Recursos Agroambientais. Agronomic Institute of Campinas, Campinas, Brazil, p. 83.

## Overview

Conducting large experiments requires experience and a lot of work, therefore, maximizing the utility of these field experimental areas is necessary. Besides conducting a diverse range of studies using the same field experiment is pivotal in scientific research for several reasons. Firstly, such an approach allows researchers to explore multiple facets of a single experiment exploring different angles. By engaging diverse research expertise and knowledge, a variety of perspectives are brought together, leading to better interpretations and broader conclusions. Secondly, the collaborative nature of such studies encourages interdisciplinary cooperation, promoting the integration of different scientific disciplines. This collaborative effort can shed light on complex ecological processes, providing a holistic view of the studied ecosystem. This approach is often seen in extensive cooperative projects. However, synthesizing findings from these varied studies into a single manuscript is of paramount importance. Often, in large-scale projects, researchers work collaboratively but publish individual papers according to their specific areas. While these papers contribute valuable insights independently, they might lack a cohesive narrative when considered together. Writing a unified paper that brings together methodologies, sampling techniques, and findings from diverse studies not only provides a consolidated resource for researchers but also offers a clear roadmap for future investigations.

In this context, the main goal of this method article is to support researchers in applying different methods and techniques to explore the soil microbiome in relation to various agronomic strategies aimed at mitigating environmental detriments, all within a single experiment. To achieve these goals, a multidisciplinary group with a diverse set of skills is necessary. The researchers should possess expertise in various fields, including soil sciences, soil fertility, crop fertilization, organic byproduct usage and recycling, efficient fertilizer utilization, nitrogen (N) loss mechanisms, greenhouse gas emissions assessment in agricultural systems, soil microbial ecology, molecular ecology, ecological genomics, soil ecology, statistics and bioinformatics. To assist these researchers, we provide guidelines and tips to design efficient experiments. Additionally, we offer ideas on the potential studies that can be conducted within a single field experiment. The methodologies applied here are versatile and can be adapted to different systems, including various plant species, both no-till and conventional tillage practices, agropastoral farming systems, and more.

**Related research article:**

Lourenço, K.S., Suleiman, A.K.A., Pijl, A., Cantarella, H., Kuramae, E.E., 2020. Dynamics and resilience of soil mycobiome under multiple organic and inorganic pulse disturbances. Science of the Total Environment, 139,173. https://doi.org/10.1016/j.scitotenv.2020.139173

Lourenço, K.S., Rossetto, R., Vitti, A.C., Montezano, Z.F., Soares, J.R., de Melo Sousa, R., do Carmo, J.B., Kuramae, E.E., Cantarella, H., 2019. Strategies to mitigate the nitrous oxide emissions from nitrogen fertilizer applied with organic fertilizers in sugarcane. Science of the Total Environment 650, 1476–1486. https://doi.org/10.1016/j.scitotenv.2018.09.037

## Ethics statements

Not applicable.

## CRediT authorship contribution statement

**Késia Silva Lourenço:** Conceptualization, Methodology, Investigation, Data curation, Visualization, Writing – original draft. **Afnan Khalil Ahmad Suleiman:** Conceptualization, Methodology, Visualization, Writing – review & editing. **Agata Pijl:** Methodology, Investigation. **Mauricio R. Dimitrov:** Methodology. **Heitor Cantarella:** Conceptualization, Methodology, Data curation, Supervision. **Eiko Eurya Kuramae:** Conceptualization, Methodology, Data curation, Supervision, Writing – review & editing.

## Declaration of competing interest

The authors declare that they have no known competing financial interests or personal relationships that could have appeared to influence the work reported in this paper.

## Data Availability

No data was used for the research described in the article.

## Appendix Supplementary materials

Image, application 1
