# Supplementary material for: Mix-method toolbox for monitoring greenhouse gas production and microbiome responses to soil amendments
Source: MethodsX. 2024 Apr 16;12:102699. doi: 10.1016/j.mex.2024.102699 (PMC11041840; doi:10.1016/j.mex.2024.102699)
Supplement: Supplementary file 1 [file mmc1.docx]

***Mix-Method Toolbox for Monitoring Greenhouse Gas Production and Microbiome Responses to Soil Amendments***

*Késia Silva Lourenço^a,b,*^, Afnan Khalil Ahmad Suleiman^a,c^, Agata Pijl^a^, Mauricio R. Dimitrov^a^, Heitor Cantarella^b,^*^,1^, Eiko Eurya Kuramae^a,d,^*^,1^*

^a^ Microbial Ecology Department, Netherlands Institute of Ecology (NIOO), Droevendaalsesteeg 10, 6708 PB Wageningen, The Netherlands

^b^ Soils and Environmental Resources Center, Agronomic Institute of Campinas (IAC), Av. Barão de Itapura 1481, 13020-902 Campinas, SP, Brazi

^c^ Soil Health group, Bioclear Earth B.V., Rozenburglaan 13, 9727 DL Groningen, The Netherlands

^d^Ecology and Biodiversity, Institute of Environmental Biology, Utrecht University, Utrecht, The Netherlands

^1^ Sharing the last authorship

*^1^Corresponding author:*

*Késia S. Lourenço, e-mail:* [*lourencokesia@gmail.com*](mailto:lourencokesia@gmail.com)*; kesia.lourenco@wur.nl*

*Eiko E. Kuramae, e-mail:* [*e.kuramae@nioo.knaw.nl*](mailto:e.kuramae@nioo.knaw.nl)*;*

*Heitor Cantarella, e-mail:* [*cantarella@iac.sp.gov.br*](mailto:cantarella@iac.sp.gov.br)

**Supplementary Figures**


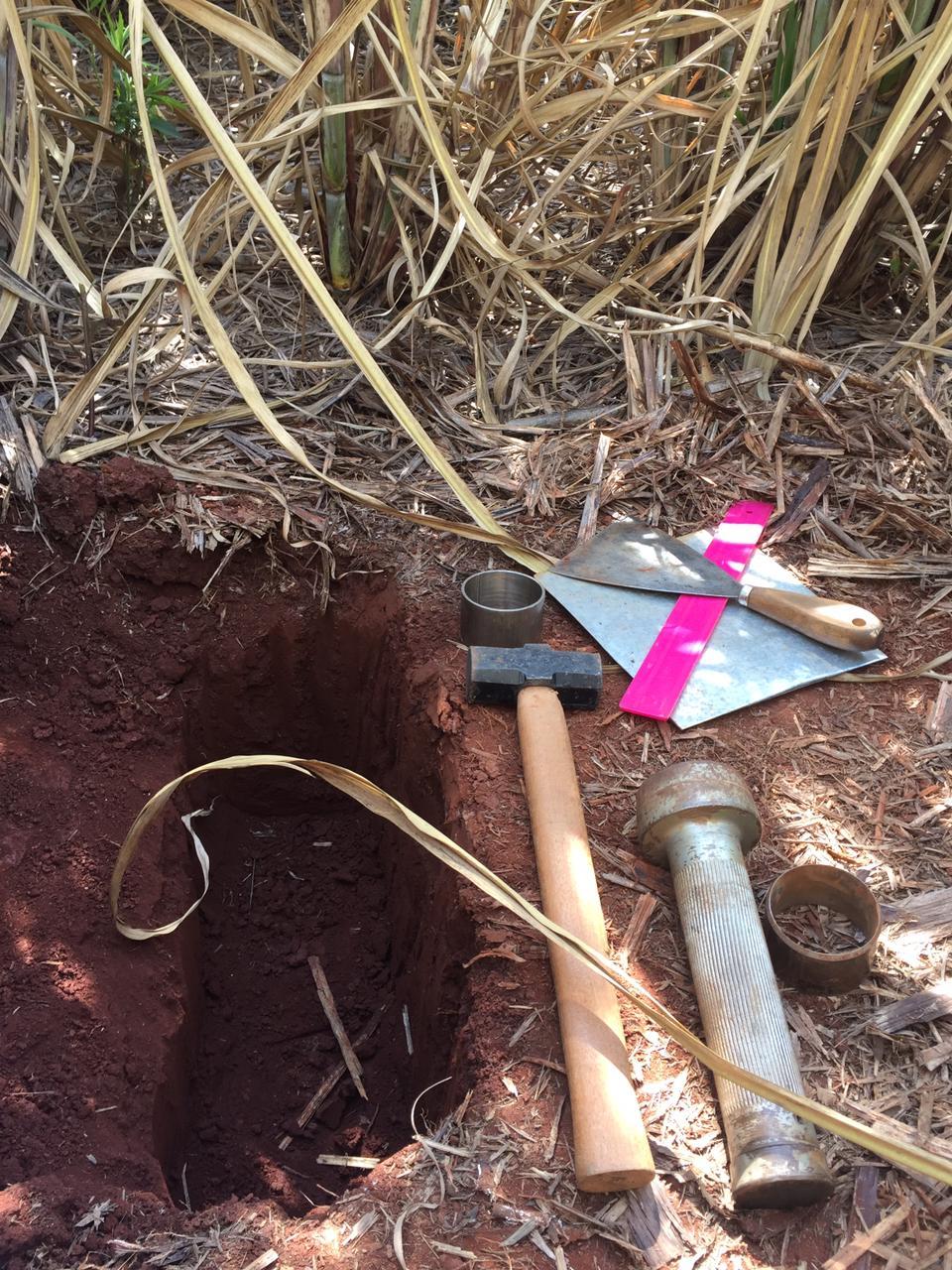


**Figure S1.** Trenches and bulk density metal ring (50 cm^3^) (The picture is just an example, Trenches with 0.5 m long, 0.2 m wide and 0.3 m deep).


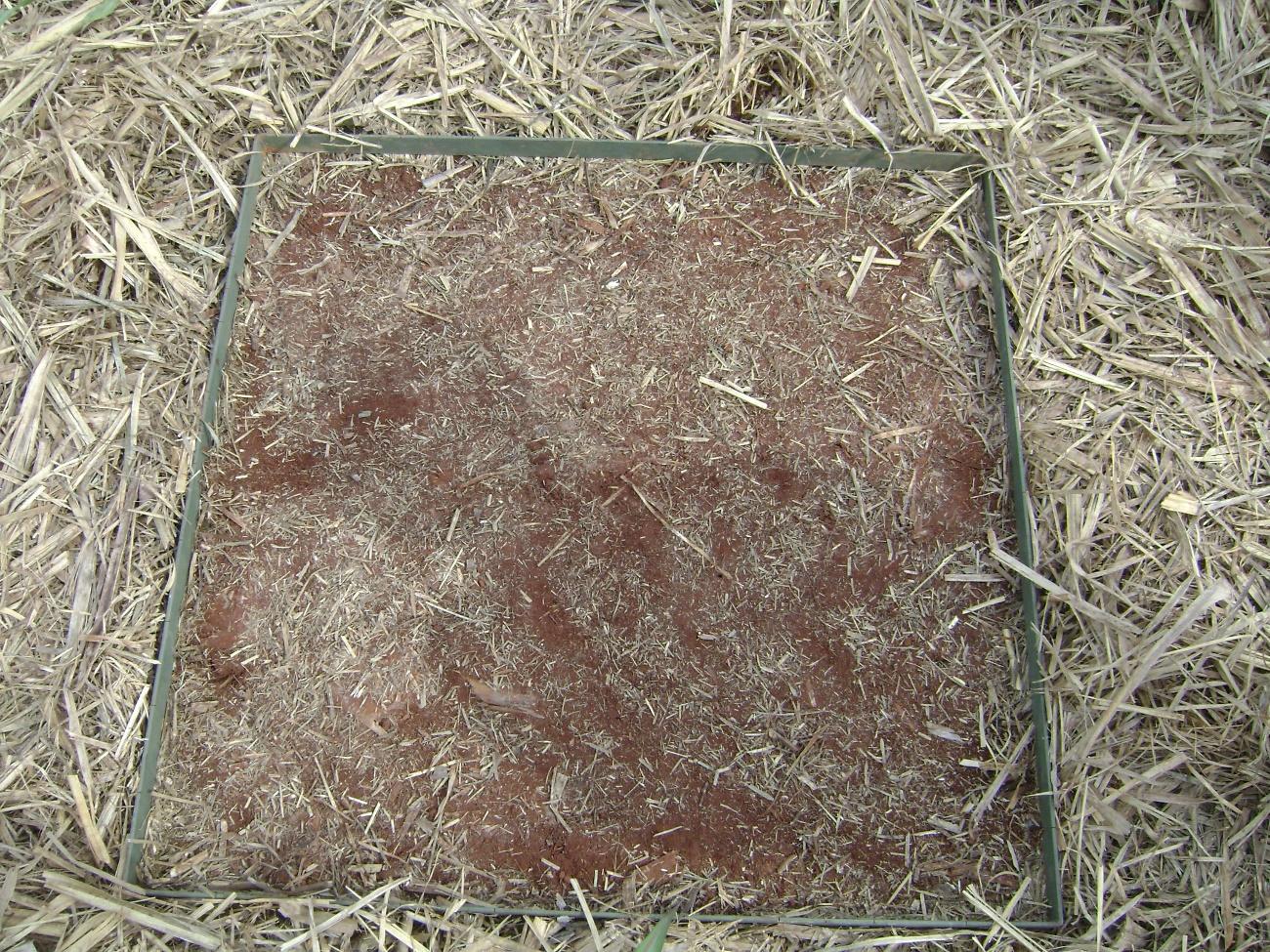


**Figure S2.** Metal frame (1 m^2^) used to determine the crop residue left on soil.


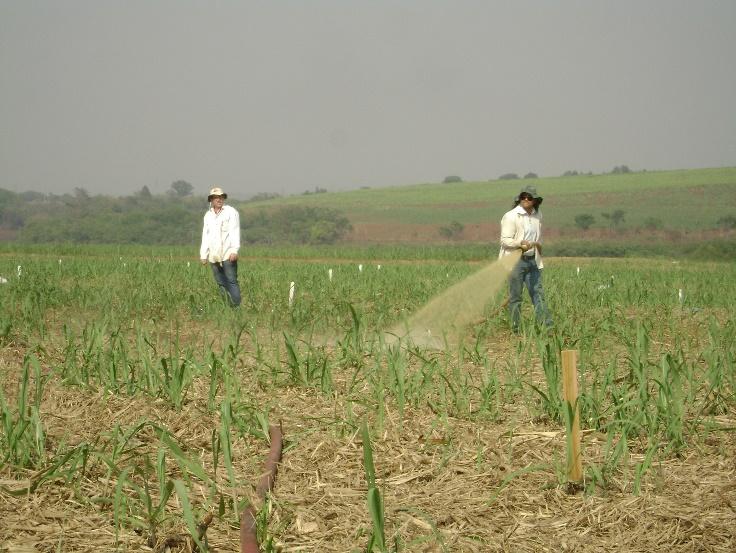

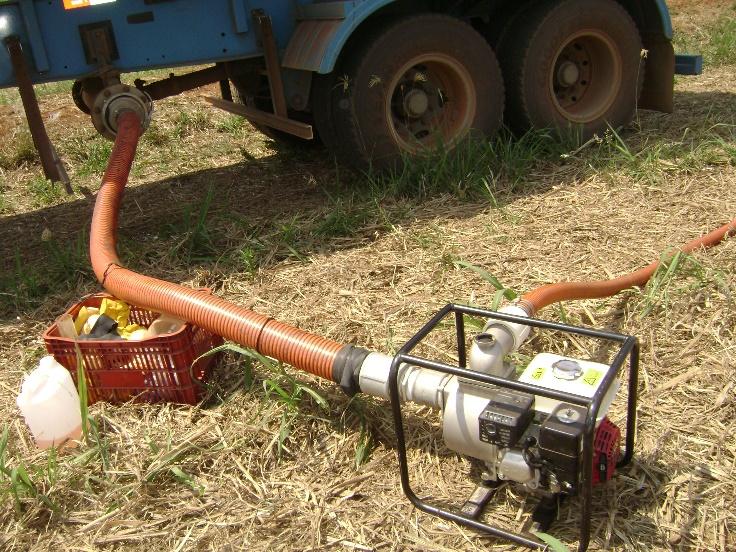


**Figure S3.** Non-concentrated vinasse been sprayed over the entire experimental plot at a rate of 100 m^3^ ha^-1^, using a motorized pump fit with a flow regulator.


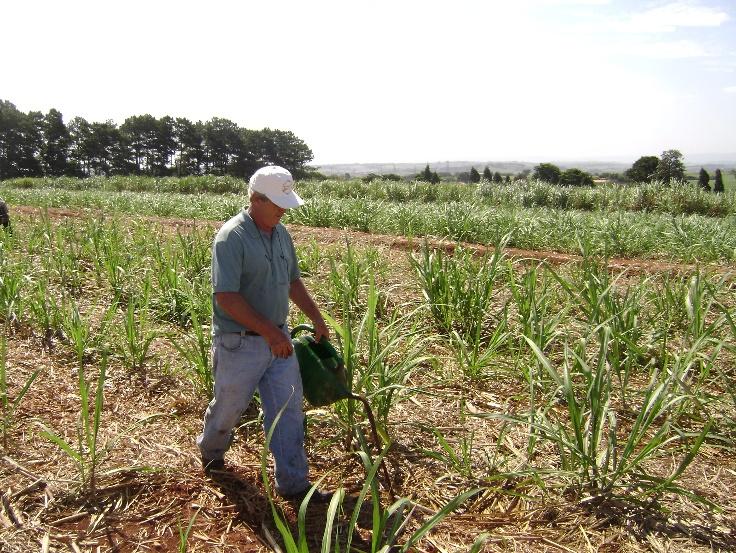

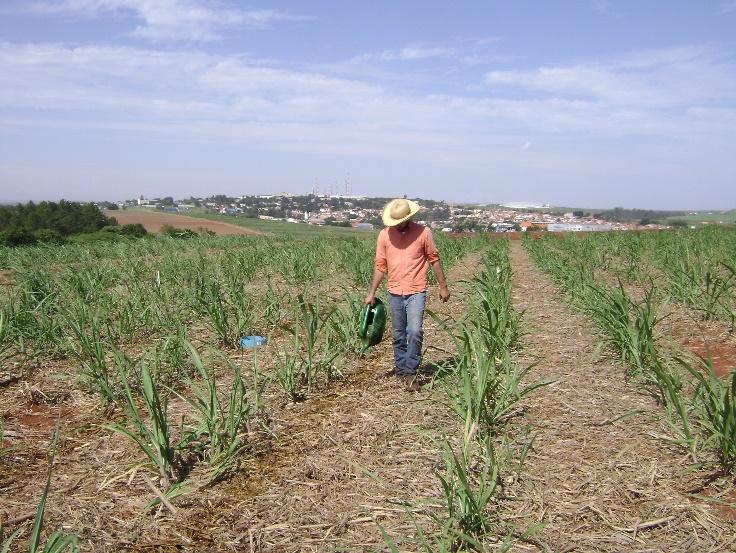


**Figure S4.** Concentrated vinasse application using calibrated plastic watering can, rate of 17.2 m^3^ ha^-1^.


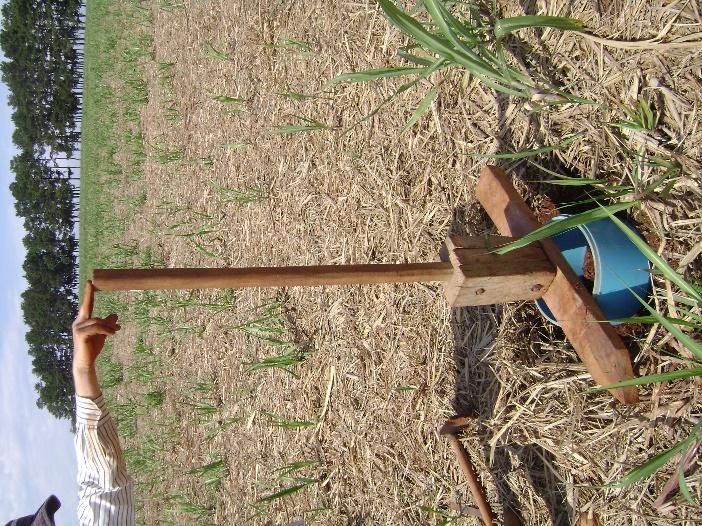

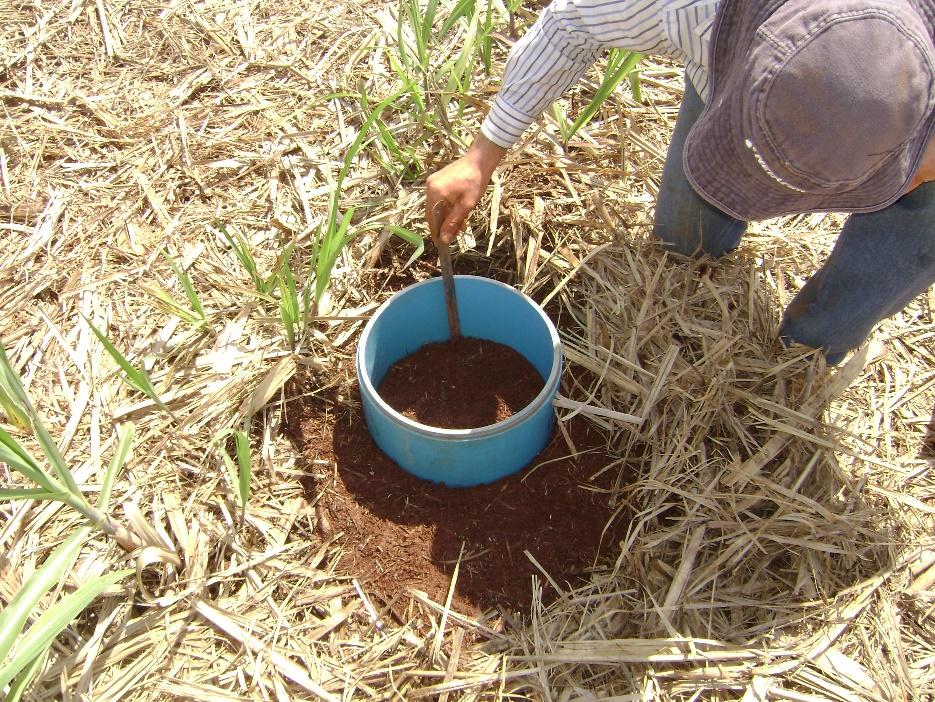


**Figure S5.** Greenhouse gas chambers (Area = 0.0707 m^2^) installation in a sugarcane field.


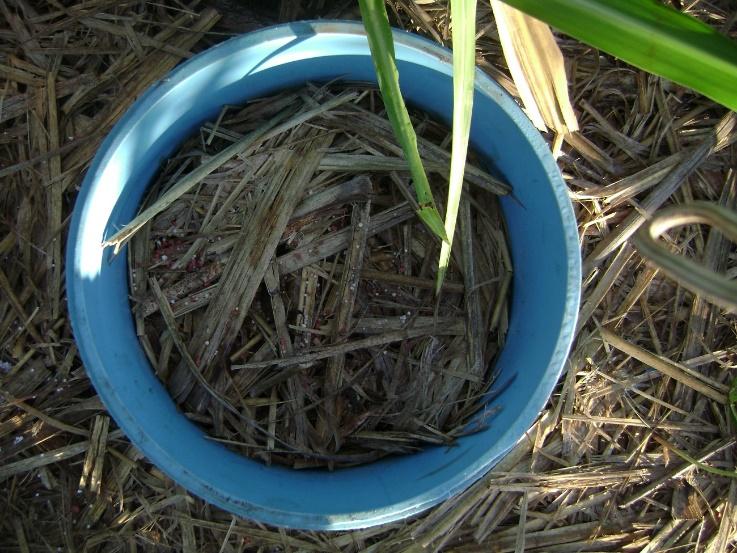

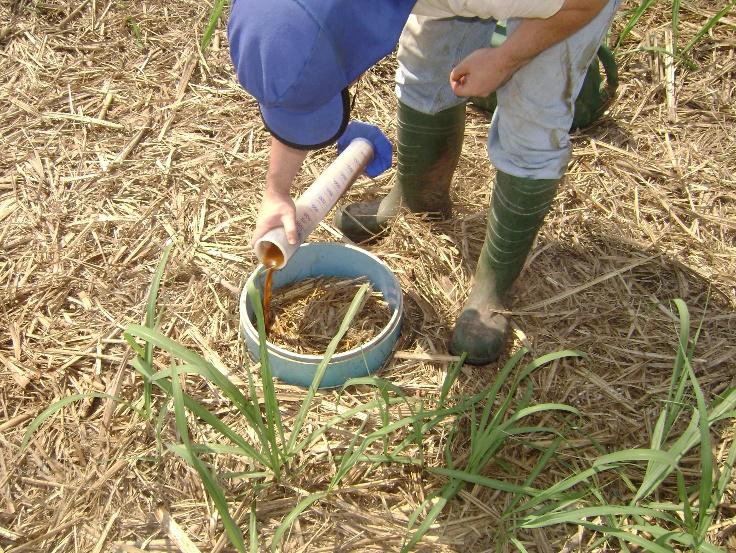


**Figure S6.** Inorganic fertilizers (N, P, and K) and vinasses application inside the greenhouse gas chambers.


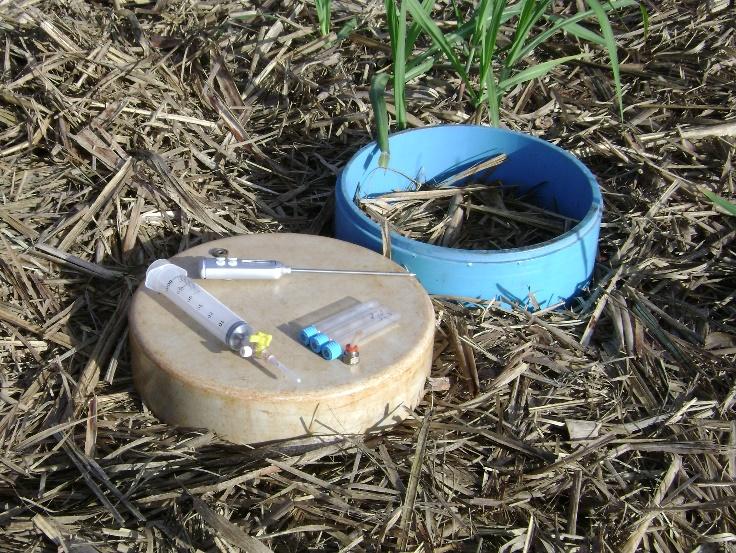

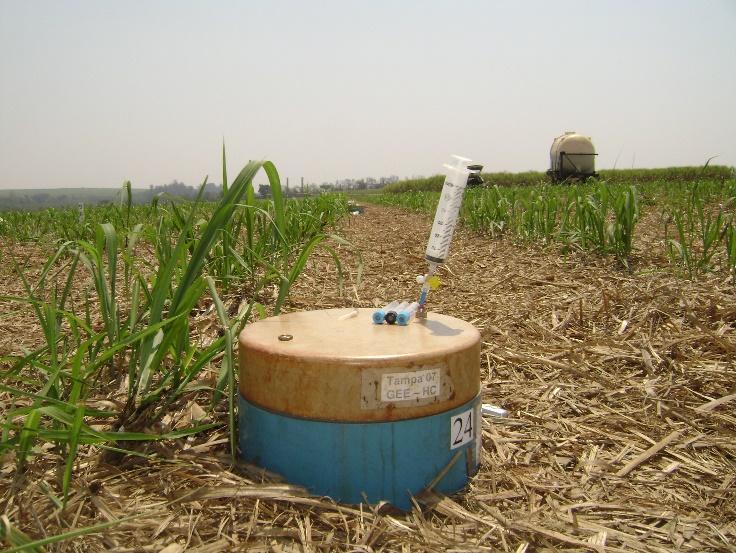


**Figure S7.** Field material needed for greenhouse gas sampling. Greenhouse gas chambers, PVC pipe cap, thermometer, plastic syringes, and pre-evacuated exetainers vials.
